# Inhibition of HIV-1 release by ADAM metalloproteinase inhibitors

**DOI:** 10.3389/fmicb.2024.1385775

**Published:** 2024-03-20

**Authors:** Joanna Ireland, Jason Segura, Genbin Shi, Julianna Buchwald, Gwynne Roth, Thomas Juncheng Shen, Ruipeng Wang, Xinhua Ji, Elizabeth R. Fischer, Susan Moir, Tae-Wook Chun, Peter D. Sun

**Affiliations:** ^1^Laboratory of Immunogenetics, National Institute of Allergy and Infectious Diseases, National Institutes of Health, Rockville, MD, United States; ^2^Center for Structural Biology, National Cancer Institute, National Institutes of Health, Frederick, MD, United States; ^3^Electron Microscopy Unit, Research Technology Branch, National Institute of Allergy and Infectious Diseases, National Institutes of Health, Hamilton, MT, United States; ^4^Laboratory of Immunoregulation, National Institute of Allergy and Infectious Diseases, National Institutes of Health, Bethesda, MD, United States

**Keywords:** HIV-1 infection of CD4 T-cells, viral release, L-selectin (CD62L), ADAM metallopeptidase domain, ADAM10 and 17, batimastat (BB-94), prinomastat, transmission electron microscopy

## Abstract

HIV-1 gp120 glycan binding to C-type lectin adhesion receptor L-selectin/CD62L on CD4 T cells facilitates viral attachment and entry. Paradoxically, the adhesion receptor impedes HIV-1 budding from infected T cells and the viral release requires the shedding of CD62L. To systematically investigate CD62L-shedding mediated viral release and its potential inhibition, we screened compounds specific for serine-, cysteine-, aspartyl-, and Zn-dependent proteases for CD62L shedding inhibition and found that a subclass of Zn-metalloproteinase inhibitors, including BB-94, TAPI, prinomastat, GM6001, and GI25423X, suppressed CD62L shedding. Their inhibition of HIV-1 infections correlated with enzymatic suppression of both ADAM10 and 17 activities and expressions of these ADAMs were transiently induced during the viral infection. These metalloproteinase inhibitors are distinct from the current antiretroviral drug compounds. Using immunogold labeling of CD62L, we observed association between budding HIV-1 virions and CD62L by transmission electron microscope, and the extent of CD62L-tethering of budding virions increased when the receptor shedding is inhibited. Finally, these CD62L shedding inhibitors suppressed the release of HIV-1 virions by CD4 T cells of infected individuals and their virion release inhibitions correlated with their CD62L shedding inhibitions. Our finding reveals a new therapeutic approach targeted at HIV-1 viral release.

## Introduction

HIV infection remains a public health threat with over 38 million infected people worldwide.^1^ Currently, the use of antiretroviral therapy (ART) remains the most effective treatment against HIV-1 infection (Martin and Siliciano, 2016). Although ART effectively controls plasma viremia in infected individuals, it is not sufficient to eradicate the virus. As a consequence, plasma viremia rebounds upon withdraw of ART in the majority of infected individuals (Eisinger and Fauci, 2018). This inability of ART to eliminate HIV in infected individuals has been attributed to the persistence of viral reservoirs that resist the antiviral treatment (Huang et al., 2018; Sengupta and Siliciano, 2018). The compounds present in ART comprise inhibitors that target the viral entry, reverse transcription, host integration, and virion maturation (De Miguel et al., 2018; Ribera, 2018). There is currently no compound that targets HIV-1 viral release. Over the years, intense efforts have been directed at the development of effective vaccines against HIV-1, resulting in a greater understanding of antibody mediated anti-viral responses and a large collection of highly potent neutralizing antibodies (Escolano et al., 2017; Bar-On et al., 2018). However, finding a protective anti-HIV vaccine remains elusive (Bar et al., 2016; Sneller et al., 2017; Andrabi et al., 2018; Gao et al., 2018; Mendoza et al., 2018; Chun et al., 2019).

We previously showed that in addition to CD4 and chemokine receptors, HIV utilizes glycan receptors, such as Siglec and L-selectin/CD62L, on target cells as adhesion receptors to facilitate efficient viral attachment and entry (Zou et al., 2011; Kononchik et al., 2018). CD62L-mediated binding to viral envelope glycans underscores preferential replication of HIV-1 in central memory CD4 T cells. Interestingly, HIV-1 infected CD4 T cells downregulated their CD62L expressions. CD62L functions to provide adhesion for lymphocyte trafficking (Von Andrian et al., 1993; Stein et al., 1999), and is a marker for central memory T cells (Sallusto et al., 2004). Upon activation or under inflammation, CD62L is known to be cleaved by cell surface transmembrane proteases, named ADAM (A disintegrin and metalloproteinase) (Le Gall et al., 2009; Wang et al., 2010). ADAM family enzymes, also known as α-secretases, are responsible for ectodomain shedding of diverse cell surface proteins, including growth factors, cytokines, receptors and adhesion molecules (Edwards et al., 2008; Saftig and Reiss, 2011). Similar to members of matrix metalloproteinase (MMP), the catalytic activities of ADAM proteases are Zn-dependent and can be inhibited by hydroxamic acid-based Zn-chelators, such as BB-94 (Batimastat) (Parsons et al., 1997; Kapp et al., 2012). This paradoxical role of CD62L, while facilitating the viral entry but detrimental to the viral exit, is best illustrated by manipulating CD62L expression in HIV-1 permissive CEM T cells. Transfection of CD62L boosted the viral infection of CEM T cells, but also resulted in the infection more sensitive to BB-94 inhibition compared to the untransfected cells. On the other hand, knocking down of endogenous CD62L reduced the viral infection, but also resulted in the loss of BB-94 inhibition to the infection (Kononchik et al., 2018). This intricate role of CD62L expression and shedding in HIV-1 infection highlights the importance of ADAM metalloproteinases in the viral transmission. The shedding of CD62L in infected CD4 T cells further depended on HIV-1 Nef induced host caspases activation as inhibition of caspases blocked L-selectin shedding and impeded the viral release (Segura et al., 2023). However, the sheddases responsible for HIV-induced CD62L shedding and the mechanism linking CD62L shedding to viral release are yet to be established (Kononchik et al., 2018). In the current study, we attempted to screen compounds that effectively inhibit CD62L shedding and HIV-1 infection, and we investigated the molecular mechanism linking CD62L shedding and HIV-1 viral release. These findings support a therapeutic approach targeting the HIV-1 viral release.

## Results

### L-selectin/CD62L shedding on PBMC depends on zinc metalloproteinases

**Figure 1:**
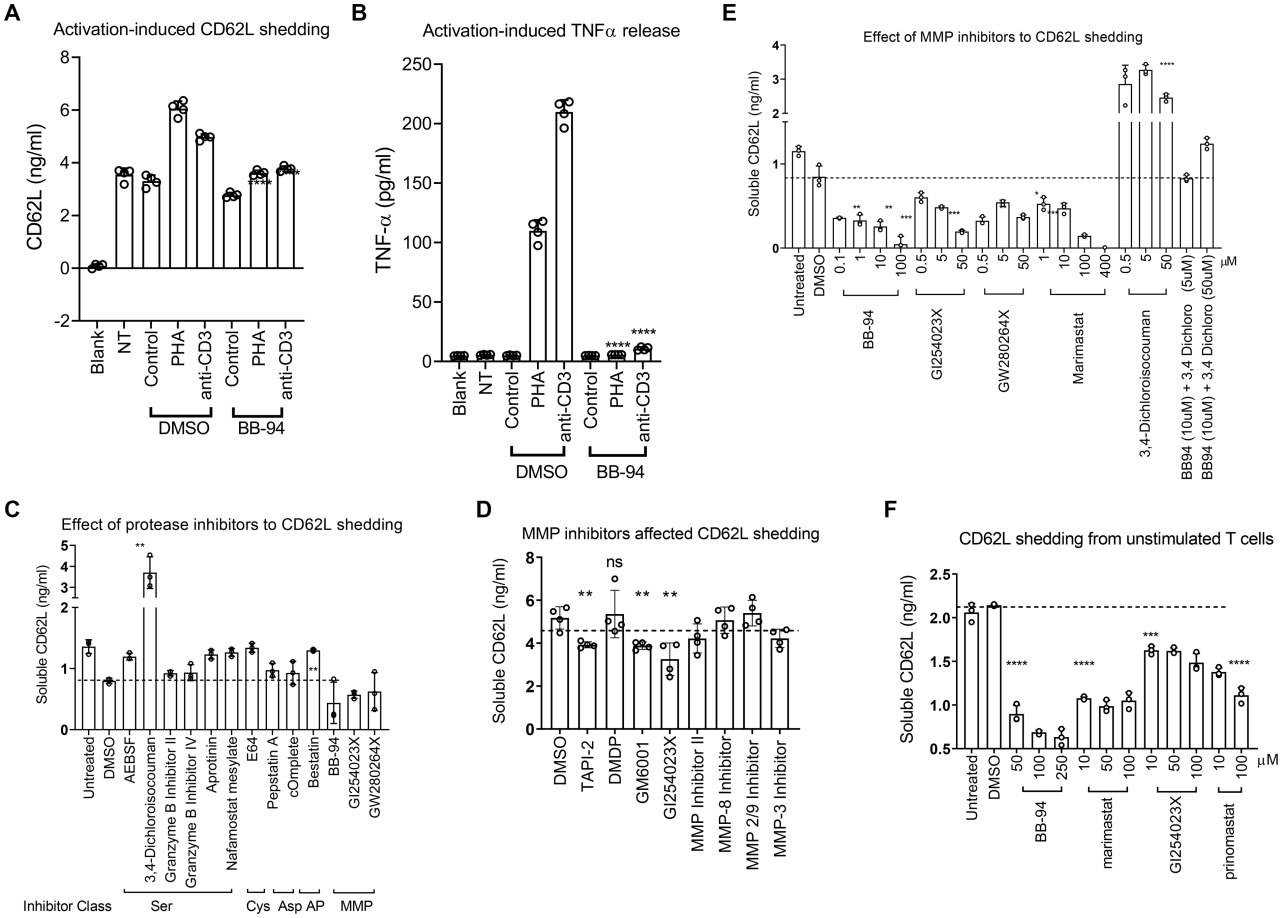
Inhibition of CD62L shedding as detected by ELISA assays. **(A,B)** CD62L shedding and TNFα release from either PHA or anti-CD3 stimulated CD4 T cells. Both soluble CD62L and TNFα released to supernatant were detected by ELISA (R&D systems). Blank, NT and control designate no cells, no treatment, and isotype control treatment, respectively. **(C–F)** Anti-CD3 stimulated **(C–E)** or unstimulated **(F)** PBMC were treated with indicated inhibitor compounds for 6 **(C,E)** or 24 **(D,F)** hours, respectively. Effect of serine-, cysteine-, aspartyl-protease inhibitors **(C)**, MMP inhibitors **(D)** as well as titration of MMP inhibitors **(E)** to the shedding of CD62L from stimulated PBMC. **(F)** Shedding of CD62L from unstimulated PBMC **(F)** in the presence of titrating amount of indicated ADAM inhibitors. All statistical analysis were performed using student’s t-test between DMSO and inhibitor treated experiments. Unless indicated with *p*-values, most treatment did not result in significant differences in the shedding of CD62L. *P*-values are * < 0.05, ** < 0.01, *** < 0.001, **** < 0.0001.

L-selectin/CD62L is a marker for central memory T cells and promotes the homing of T cells to lymph nodes (Von Andrian et al., 1993; Sallusto et al., 2004). We previously showed that L-selectin shedding facilitated HIV-1 release (Kononchik et al., 2018). However, the proteases responsible for the viral-induced CD62L shedding remain to be defined. CD62L shedding is mainly studied in the context of neutrophil activation and has been shown to involve ADAM10 and ADAM17 metalloproteinases (Le Gall et al., 2009; Wang et al., 2010; Saftig and Reiss, 2011). Like neutrophils, activated CD4 T cells shed CD62L and release TNFα that can be detected by ELISA (Figures 1A,B). The release of both TNFα and soluble CD62L were inhibited by batimastat (BB-94), a matrix metalloproteinase inhibitor that inhibits both ADAM10 and 17 (Saftig and Reiss, 2011), suggesting the involvement of ADAM enzymes in the shedding of CD62L on CD4 lymphocytes. To further characterize the enzymes responsible for T cell shedding of CD62L and thus facilitate the design of protease specific inhibitors as potential antiviral reagents, we measured shedding of CD62L by ELISA in the presence of various protease inhibitors. As T cells express other proteases (Kido et al., 1990), we systematically evaluated the inhibition of CD62L shedding in both anti-CD3 stimulated and non-stimulated cell cultures by serine-, cysteine-, aspartyl-proteases as well as metalloproteinases inhibitors (Table 1). Stimulated or unstimulated peripheral blood mononuclear cells (PBMC) were treated with inhibitors specific against various proteases for 6–24 h. None of the serine-, cysteine-, or aspartyl-protease inhibitors affected the release of soluble CD62L from either stimulated or naïve PBMC (Figure 1C; Supplementary Figure 1A; Table 1). We then evaluated ~20 additional known inhibitors of Zn-dependent metalloproteinases for their ability to inhibit CD62L shedding from stimulated PBMC. Most of the metalloproteinase inhibitors did not inhibit CD62L shedding, except TAPI-2, GM6001, GI254023X, BB-94 and prinomastat (Figure 1D; Table 1; Le Gall et al., 2009; Moller-Hackbarth et al., 2013; Woods and Padmanabhan, 2013). These compounds target Zn-dependent metalloproteinases and are known to inhibit ADAM10 and 17 (Parsons et al., 1997; Hooper and Turner, 2002; Woods and Padmanabhan, 2013). Further dose titration of these compounds showed that the reductions in CD62L shedding from both stimulated and unstimulated T cells were dependent on the inhibitor doses with minimum cytotoxicity between 20–100 μM compound concentrations (Figures 1E,F; Supplementary Figure 1B). Interestingly, 3,4-dichloroisocouman (or 3,4-dichloro for short), a serine protease inhibitor enhanced CD62L shedding and this shedding enhancement was blocked by BB-94 (Figure 1E).

**Table 1:** Compounds used in ELISA to inhibit CD62L shedding from CD4 T cells.

	Protease specificity	Concentration (μM)	Shedding inhibition	
			Stimulated	Non-stim.
BB-94, Batimastat	MMP-1,2,7,9,13,14, ADAM17,10	10	++	++
Marimastat	MMP-1,2,7,9,14, ADAM17,10	10	++	++
GI254023X	ADAM10	10	++	+
GW280264X	ADAM17, 10	10	+/−	+/−
GM6001	MMP-1,2,7-9,12-14, ADAM17	10	+	+
UK-356618	MMP-3	10	−	−
ARP 101	MMP-2	10	+/−	
CP-471474	MMP-2,3,9,13	10	−	
ONO-4817	MMP-2,3,8,9,12,13	100	+/−	+
NSC405020	PEX domain of MMP-1	10	−	
PD166793	MMP-2,3	10	−	
Prinomastat	MMP-2,3,9,13,14	10	++	++
SB-3CT	MMP-2,9	10	−	
5-m-5-p	MMP-2,3	10	−	
4-m-GPDLA	MMP-1	10	−	
5-Indolylb	MMP-13	10	−	
DMDP	MMP-1	10	−	
Doxycycline	MMP-1	10	−	
NNGH	MMP-3	10	−	
CP-101537	MMP	10	−	
MMP inhibitor II	MMP	10	−	−
MMP-8 inhibitor	MMP-8	10	−	
TAPI-0,-1,-2	ADAM17, 10	10	++	
Bestatin	Aminopeptidase	10	−	
3,4-DCIC	Serine proteases	10	−	
GrzB I	Granzyme B	10	−	
GrzB II	Granzyme B	10	−	
GrzB IV	Granzyme B	10	−	
Leupeptin	Serine, cysteine proteases	10	−	
AEBSF	Serine proteases	10	−	
Aprotinin (BPTI)	Serine proteases	10	−	
Nafamostat	Serine proteases	10	−	
cOmplete PI cocktail	Ser, Cys, Asp proteases	10	−	
PMSF	Serine proteases	10	−	
Cystatin	Cysteine proteases	10	−	
E64	Cysteine proteases	10	−	
Iodoacetamide	Cysteine proteases	10	−	
Pepstatin A	Aspartyl proteases	10	−	

++, +, +/− and – represents compounds with >50%, 20–50, <20 and 0% inhibition.

UK-356618: (2R)-N1-[(1S)-2,2-Dimethyl-1-({[(1R)-1-phenylethyl]amino}carbonyl)propyl]-2-{3-[(3-methyl-4-phenyl)-phenyl]propyl}-(N4-hydroxy)butanediamide; ARP 101: (2R)-2-[([1,1′-Biphenyl]-4-ylsulfonyl)(1-methylethoxy)amino]-N-hydroxy-3-methyl-butanamide, (R)-N-Hydroxy-2-(N-isopropoxybiphenyl-4-ylsulfonamido)-3-methylbutanamide; CP-471474: 2-[[[4-(4-Fluorophenoxy)phenyl]sulfonyl]amino]-N-hydrox y-2-methylpropanamide; PD166793: (S)-2-(4′-Bromo-biphenyl-4-sulfonylamino)-3-methyl-butyric acid hydrate; 5-m-5-p: 5-methyl-5-phenylhydantoin; 4-m-GPDLA: 4-aminobenzoyl-Gly-Pro-D-Leu-ala hydroxamic acid; 5-indolylb: 5-indolylboronic acid; DMDP: Dichloromethylenediphosphonic acid disodium salt; 3,4-DCIC: 3,4-Dichloroisocouman; GrzB I, II, IV: granzyme B inhibitor I, II, IV; NSC405020: 3,4-Dichloro-N-(1-methylbutyl)-benzamide, 3,4-Dichloro-N-(pentan-2-yl)benzamide; CL-82198: N-[4-(4-Morpholinyl)butyl]-2-benzofurancarboxamide; NNGH: N-Isobutyl-N-(4-methoxyphenylsulfonyl)glycyl hydroxamic acid; CP-101537: N-(1a,5a,6a)-3-Azabicyclo[3.1.0]hex-6-yl-carbamic acid 1,1-dimethylethyl ester; PI cocktail: a mixture of AEBSF, Aprotinin, Bestatin, E-64, Leupeptin, Pepstain A.

### Inhibition of ADAM10 and 17 enzymatic activities by L-selectin shedding inhibitors

**Figure 2:**
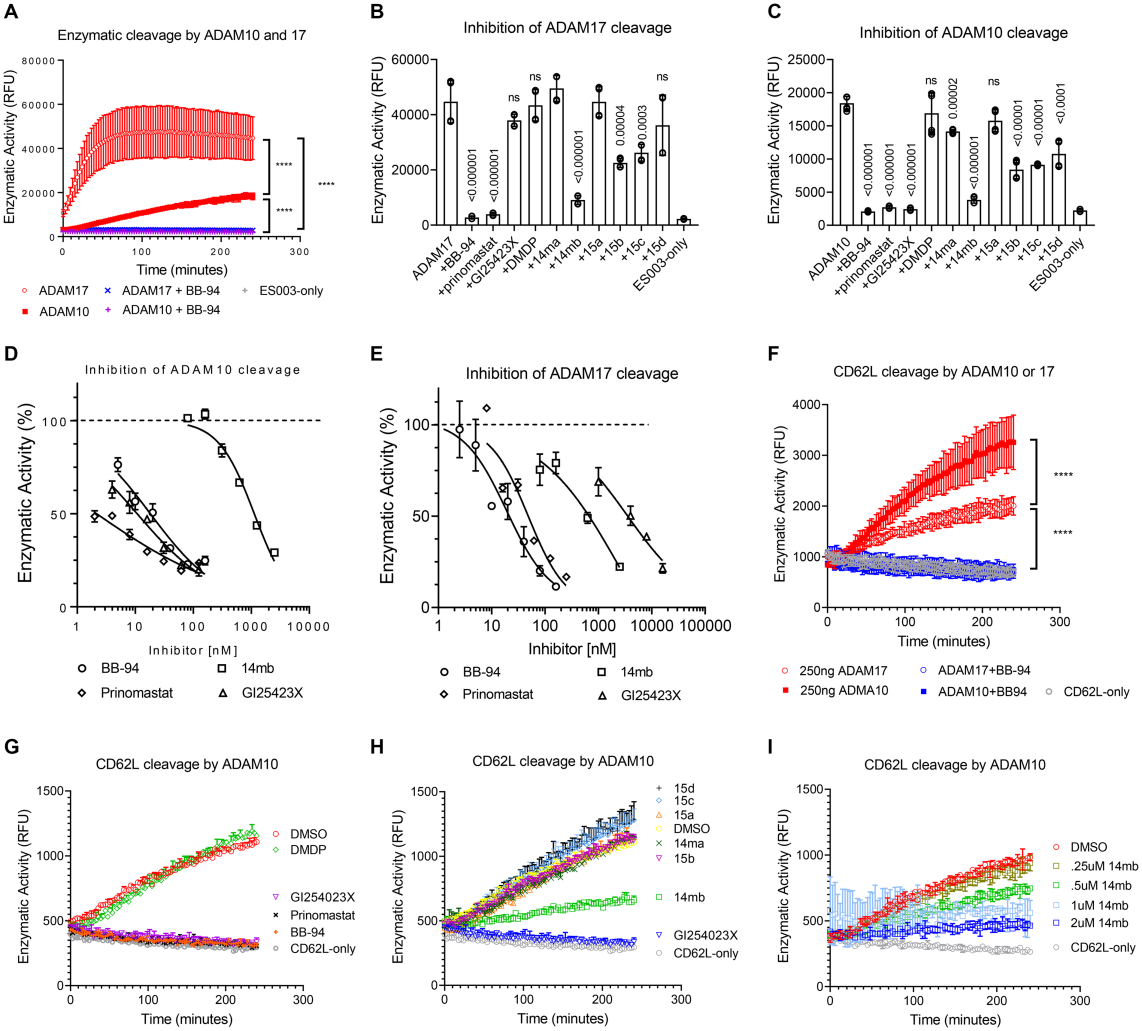
Inhibition of ADAM10 and 17 proteolytic activities using a kinetic enzymatic cleavage assay. **(A)** Kinetic cleavage of a fluorogenic TNF-α substrate peptide ES003 by recombinant ADAM10 or 17 (R&D systems, Inc) in the presence and absence of 2 μM BB-94. In the presence of BB-94, the kinetic ES003 cleavage curves by ADAM10 and 17 are indistinguishable from that of ES003-only curve without the enzyme. **(B,C)** Inhibition of ADAM17 **(B)** or ADAM10 **(C)** cleavage of ES003 by various compounds at 2 μM concentration. All statistics are calculated between no inhibitor and respective inhibitor data sets using multiple *t*-tests with *p*-values indicated above their data. The data are a representative of two repeated experiments. **(D,E)** Inhibition of ADAM10 **(D)** and ADAM17 **(E)** enzymatic activities by titrating amount of BB-94, GI254023X, prinomastat, and 14mb. The compound concentrations are indicated on the *x*-axis. The enzymatic activities measured at 4-h time point of the reactions are normalized against zero compound condition and plotted on the *y*-axis. The inhibition curves are fitted with non-linear regression with inhibition constants (Ki) for each compound listed in Table 2. **(F)** Enzymatic cleavage of a fluorogenic CD62L peptide by recombinant ADAM10 or 17. The CD62L cleavage curves in the presence of 2 μM BB-94 are indistinguishable with that of fluorogenic peptide CD62L-only without the enzyme. **(G,H)** Inhibition of ADAM10 cleavage of CD62L substrate peptide by various compounds at 2 μM concentration. **(I)** Inhibition of ADAM10 cleavage of CD62L by titrating amount of 14mb. *P*-values are ****<0.0001.

To address the potency and specificity of CD62L shedding inhibitors against ADAM10 and 17, we carried out an enzymatic cleavage assay using recombinant ADAM10 and 17 to cleave a fluorogenic TNF-α substrate in the presence of 2 μM individual compounds (Figures 2A–C; Supplementary Figure 2). While the broad specificity metalloprotease inhibitors BB-94 and prinomastat inhibited both ADAM10 and 17, an MMP-1 specific inhibitor, Dichloromethylenediphosphonic acid disodium salt (DMDP) inhibited neither ADAM10 nor 17 enzymatic activities (Supplementary Figures 2A,D), consistent with DMDP’s lack of CD62L shedding inhibition (Figures 2B,C; Table 1). As expected, GI254023X, a ADAM10 specific compound, preferentially inhibited the enzymatic activity of ADAM10 compared to ADAM17 (Figures 2B–E; Table 2). In addition, we also synthesized two 3-Cl-phenylpyrrolidine tartrate diamide analogs, 14 m and 15, that were developed specifically against ADAM17 to further investigate the involvement of ADAM17 in HIV infection (Li et al., 2010). Compounds 14 m and 15 differ only by one methyl-group (Supplementary Figure 3A; Li et al., 2010). The chemical synthesis resulted in 2 racemic isomers for compound 14 m (14ma and 14mb) and 4 racemic isomers for compound 15 (15a, b, c, d). Between the two compound 14 m isomers, 14mb exhibited better inhibition to both ADAM10 and ADAM17 enzymatic cleavage of the fluorogenic peptide (Supplementary Figures 2B,E; Figures 2B,C). In contrast, the best of compound 15 isomers, 15b, only marginally inhibited the enzymatic cleavage (Supplementary Figures 2C,F). Further titration inhibition resulted in inhibition constants (Ki) for BB-94 of 18 nM against both ADAM17 and 10, for prinomastat of 48 nM and 2 nM against ADAM17 and 10, respectively, and 0.5–1.1 μM for 14mb against ADAM17 and 10, respectively (Table 2; Figures 2D,E).

**Table 2:** Inhibition of ADAM10 and 17 enzymatic cleavage.

	Inhibition constant Ki x10^−6^M	
Inhibitor	ADAM17	ADAM10
BB-94	0.019 ± 0.007	0.018 ± 0.002
prinomastat	0.048 ± 0.036	0.002 ± 0.0004
GI25423X	3.6 ± 0.38	0.011 ± 0.009
14mb	2.7 ± 9.7	1.13 ± 0.073

We then examined ADAM10 and 17 cleavage of a fluorogenic CD62L peptide substrate. While ADAM17 cleaved TNF-α peptide better than ADAM10 (Figure 2A), the enzymes showed reverse activities against CD62L (Figure 2F), suggesting ADAM10 may be more effective in shedding of CD62L than ADAM17. Similar to the cleavage of TNF-α peptide, BB-94, GI254023X and prinomastat but not DMDP completely suppressed ADAM10 cleavage of CD62L at 2 μM compound concentration (Figure 2G). In comparison, the tartrate-based compounds (14ma, mb, 15a,b,c,d) were significantly less potent in inhibiting ADAM10 (Figure 2H), with the best inhibitory compound, 14mb, displaying a Ki of 1.6 μM against ADAM10 cleavage of CD62L (Figure 2I; Supplementary Figures 3B,C).

### Inhibition of HIV-1 infection requires suppressing both ADAM10 and 17

**Figure 3:**
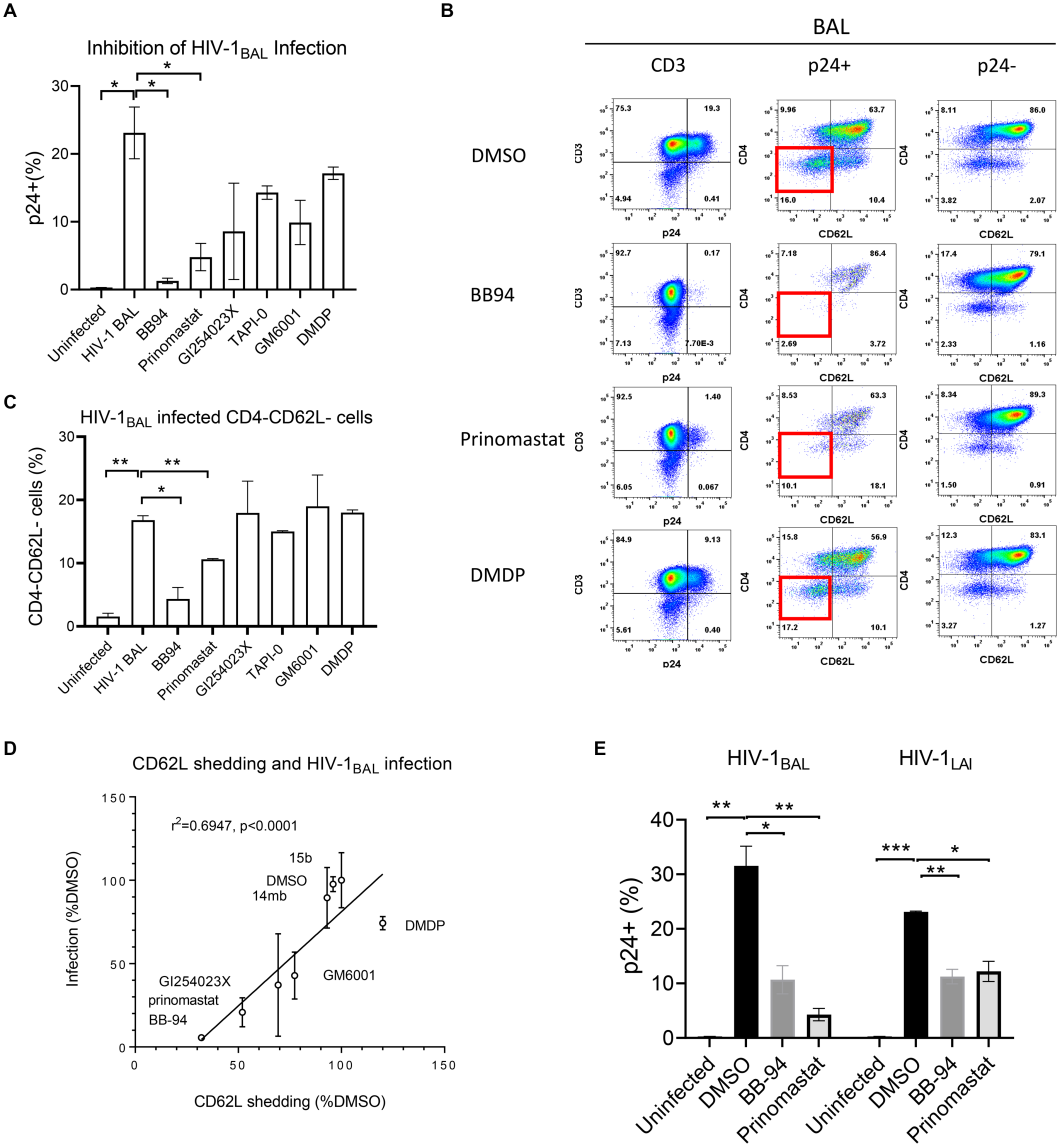
Effect of MMP inhibitors to HIV-1 infection of PBMC. **(A)** Bar diagram showing the inhibition of R5 tropic HIV-1_BAL_ infection by MMP and ADAM10/17 inhibitors. All compounds were at 100 μM concentration except GI254023X which was at 250 μM. The infection is measured by percentage of intracellular p24 staining of CD3^+^ T cells as shown in the panel **(B)** of the figure. **(B)** FACS analyses of HIV-1 induced loss of CD4 and CD62L in infected CD4 T cells in the presence of 100 μM BB-94, 80 μM prinomastat, 100 μM DMDP or control DMSO. Left panel shows the viral infection levels by intracellular p24 staining (% p24^+^ population) in CD3^+^ cells. The p24^+^ (middle panel) and p24^−^ (right panel) CD3^+^ populations are further separated by CD62L and CD4 expressions. The quadrant corresponding to CD4^−^/CD62L^−^ cells is highlighted in red box in the p24^+^ populations. **(C)** Bar diagram showing the accumulation of CD4^−^/CD62L^−^ population in HIV-1 infected cells in the presence of various inhibitors. **(D)** Correlation between CD62L shedding and HIV-1 infection in the presence of various compounds. The *x*-axis represents the CD62L shedding level detected from each compound normalized against that of DMSO. The *y*-axis represents the relative infection level in the presence of individual compounds with respect to DMSO. A linear regression resulted a straight line with r-square for linearity goodness of fit being 0.6947 and a *p*-value of <0.0001 when compared to null hypothesis (no correlation). **(E)** Comparison in inhibition of R5 and X4 tropic HIV-1 infections of PBMC by BB-94 and prinomastat. The PBMC for the infections were from the same donor. All compounds were used at the same concentrations as in panel **(A)**. Unless indicated with *p*-values, the treatments did not result in significant differences from their controls. *P*-values are *<0.05, ** <0.01, *** <0.001.

To investigate the contribution of individual ADAM to HIV-1 infection, we infected CD8-depleted PBMC with a replication competent R5 tropic HIV-1_BAL_ in the presence of either ADAM10 or 17 specific or broad specificity inhibitors and analyzed the intracellular p24 levels on day 7 of post infections (Figure 3). Both BB-94 and prinomastat, inhibited HIV-1_BAL_ infections without significant impact to cell viabilities (Figures 3A,B; Supplementary Figure 4A). As GI254023X distinguished ADAM10 and 17 enzymatic activities (Figures 2D,E), we investigated the effect of their specific inhibition to HIV-1 infection using either GI254023X or TAPI-0, a known ADAM17 inhibitor. In comparison, individual ADAM10 and 17 inhibitor, GI254023X and TAPI-0, respectively, fail to significantly suppress the viral infections, suggesting the involvement of both ADAM10 and 17 in HIV-1 infections. Importantly, DMDP, an MMP-1 inhibitor that did not inhibit ADAM10 and 17 enzymatic activities nor CD62L shedding (Figures 1E, 2B,C), failed to inhibit HIV-1_BAL_ infections (Figures 3A,B). To address the mechanistic connection between ADAM-mediated CD62L shedding and HIV-1 infection, we examined the loss of CD62L expression in infected (p24+) T cells and its dependence on ADAM inhibitions. Upon infection, HIV-1 down regulated cell surface expression of CD62L, as evident from the preferential accumulation of CD4 and CD62L double negative T cells in the p24^+^ compared to the p24^−^ populations or uninfected samples (Figure 3B; Supplementary Figure 4B; Kononchik et al., 2018). The presence of BB-94 or prinomastat reduced the accumulation of infected CD4^−^/CD62L^−^ T cells in the R5 HIV-1 infections, suggesting the loss of CD62L expression in infected T cells is ADAM dependent. Similar to their lack of inhibitions to the p24 levels, the individual ADAM10 or 17 inhibitors, GI254023X or TAPI-0, respectively, did not inhibit the accumulation of CD4^−^/CD62L^−^ T cells compared to BB-94 and prinomastat (Figure 3C), consistent with the involvement of both ADAM10 and 17 in down-regulation of CD62L. Importantly, the MMP-1 inhibitor, DMDP, failed to prevent the loss of CD62L on HIV-1_BAL_ infected cells (Figures 3B,C). Indeed, the effect of these inhibitors to HIV-1_BAL_ infection correlated with their ability to inhibit CD62L shedding (Figure 3D). Interestingly, when cells were treated with BB-94 on day 3 and 5 after the initiation of the infection, the compound showed a progressive reduction in its inhibition (Supplementary Figure 4C). This is likely due to the presence of multi-round replications of HIV-1_BAL_ during the experiment (Murray et al., 2011). In addition, a serine protease inhibitor, 3,4-dichloroisocouman, significantly increased soluble CD62L concentration in treated media compared to the control (Figure 1E; Supplementary Figure 1). The compound, however, did not enhance HIV-1 infection (Supplementary Figure 4D), suggesting either the viral-induced CD62L shedding is sufficient for its release or 3,4-dichloroisocouman did not affect CD62L shedding but merely reduced the degradation of soluble CD62L in treated cell culture supernatant. To address if X4 tropic HIV-1 infection also depended on CD62L shedding, we infected CD8-depleted PBMC with a replication competent X4 tropic HIV-1_LAI_ or the R5 tropic HIV-1_BAL_ in the presence of BB-94 or prinomastat. Both compounds inhibited the X4 tropic HIV-1_LAI_ infections similar to their inhibitions to the R5 tropic HIV-1_BAL_ (Figure 3E), suggesting ADAM10 and 17 are also involved in X4 tropic HIV-1 infections.

**Figure 4:**
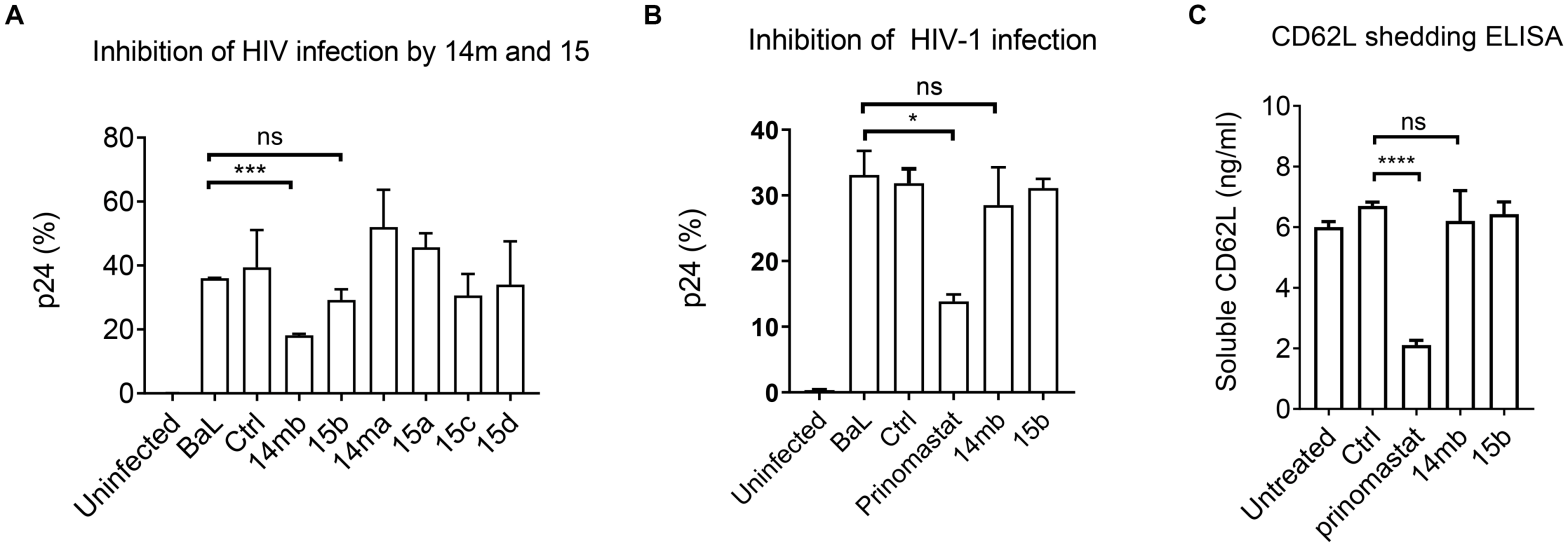
Inhibition of HIV-1_BaL_ infection by 3-Cl-phenylpyrrolidine tartrate diamide analogs 14 m and 15. **(A)** Effect of 14 m and 15 to HIV-1_BaL_ infection of PBMC as measured by intracellular p24-staining. Compounds 14 m and 15 were synthesized as 14ma, 14mb, 15a,15b,15c,15d racemers. Among them 14mb and 15b are the most active racemers of compound 14 m and 15. All compounds were used at 100uM in ELISA and infections. **(B,C)** Comparison of ADAM17 specific inhibitors 14mb and 15b with prinomastat in inhibiting HIV infection **(B)** and CD62L shedding in cells from the same donor **(C)**. *P*-values are *<0.05, ***<0.001, ****<0.0001.

Although 3-Cl-phenylpyrrolidine tartrate diamide analogs were developed as ADAM17 inhibitors, two of the isoforms,14mb and 15b inhibited ADAM10 and 17 enzymatic activities at 2–10 μM concentrations (Figure 2; Table 2). They are 50–100 times less potent compared to 20–50 nM concentrations of BB-94 and prinomastat. Despite with dual ADAM inhibitory specificities, 14mb only partially inhibited HIV-1_BAL_ infection of PBMC and the loss of CD62L expression from some donors (Figures 4A,B; Supplementary Figure 4E). When 14mb and 15b failed to inhibit HIV-1 infection of PBMC, they also failed to suppress CD62L shedding (Figure 4C), suggesting both potency and broad specificity are needed to suppress HIV-1 infection. Consistently, those tartrate analogs failed to inhibit ADAM10 digestion of CD62L, including 14ma, 15a,b,c, also failed to suppress HIV-1_BAL_ infection (Figures 2H, 4A). Together, our data showed that both R5 and X4 HIV-1 infections as well as the loss of CD62L expression on infected T cells are inhibited by broad spectrum metalloproteinase inhibitors, suggesting HIV-1 infections depend on ADAM-mediated shedding of CD62L.

### CD62L shedding inhibitors are functionally distinct from antiretroviral compounds

**Figure 5:**
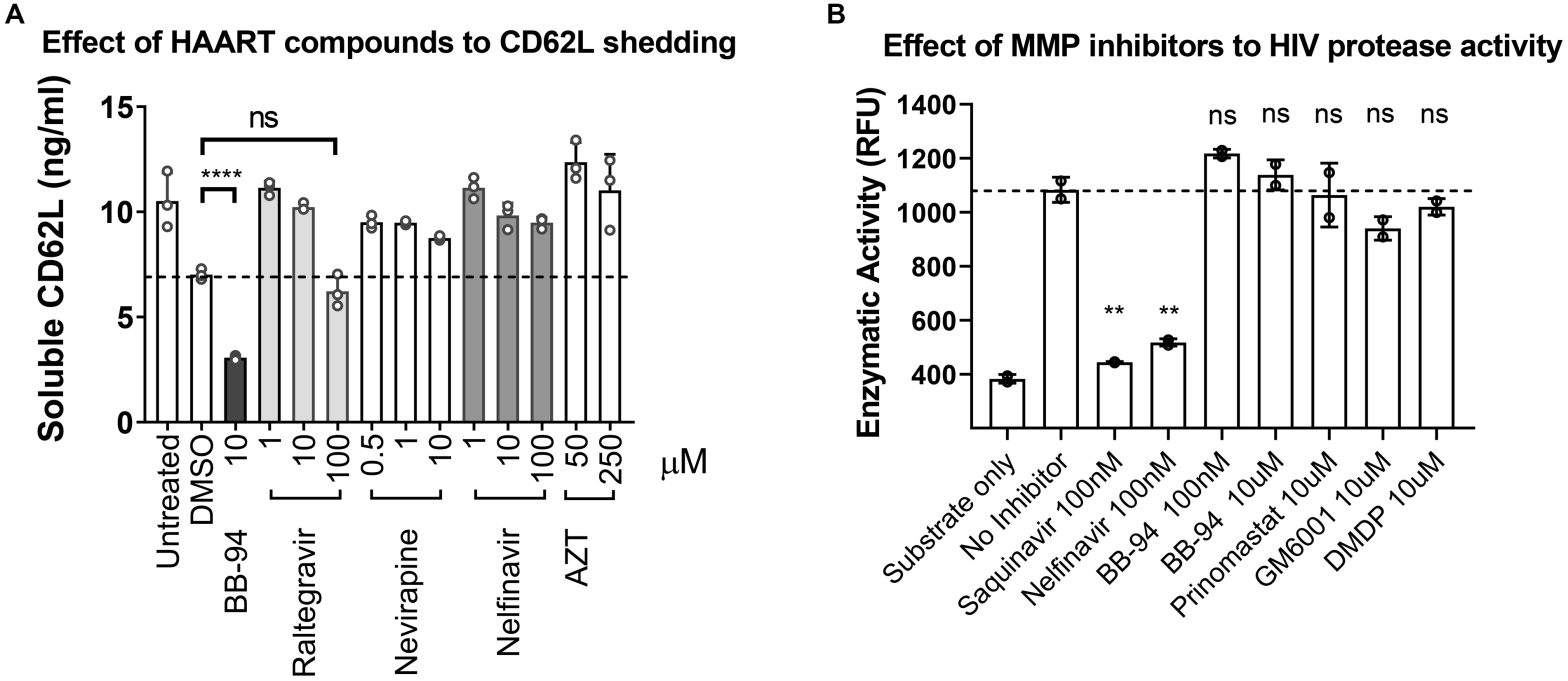
HAART compounds and CD62L shedding inhibitors do not overlap. **(A)** Stimulated PBMC were treated with titration concentrations of HAART compounds, including raltegravir, nevirapine, nelfinavir and AZT. Soluble CD62L in the supernatant were measured by ELISA. In contrast to BB-94, HAART compounds did not inhibit CD62L shedding from stimulated PBMC in the concentration range tested. **(B)** Effect of MMP inhibitors, including BB-94, prinomastat, GM6001 and DMDP, on the enzymatic activity of HIV-1 protease. HIV protease activity was measured by an enzymatic cleavage assay using recombinant viral protease to cleave a fluorescent substrate peptide in the presence of viral protease inhibitors (saquinavir or nelfinavir), or BB-94, prinomastat, GM6001 or DMDP at indicated concentrations. *P*-values are **<0.01, ****<0.0001.

Currently, antiretroviral therapy includes several classes of compounds targeted at HIV entry, reverse transcriptase, integrase, and the viral protease (Lu et al., 2018). ADAMs are Zn^2+^-dependent metalloproteinases distinct from HIV protease, a retroviral aspartyl protease. Metalloproteinase inhibitors that target CD62L shedding constitute a potential new class of antiviral compounds. It is not clear, however, if the ART compounds affect CD62L shedding. To assess the potential overlap between ART compounds and CD62L shedding inhibitors, we evaluated the ability of HIV protease inhibitor (nelfinavir), reverse transcriptase inhibitor (nevirapine, AZT), and integrase strand transfer inhibitor (raltegravir) to inhibit L-selectin shedding from activated PBMCs (Vera et al., 2015). None of these ART compounds significantly inhibited CD62L shedding at their effective dose concentrations compared to BB-94, as measured by ELISA (Figure 5A; Supplementary Figures 5A–C). Conversely, to address if CD62L shedding inhibitors suppressed HIV-1 protease activity, we carried out recombinant HIV protease cleavage of its fluorogenic substrate peptide in the presence of various MMP or viral protease inhibitors (Vella, 1994). While the known viral protease inhibitors, saquinavir and nelfinavir, both suppressed the substrate cleavage by HIV protease, none of the MMP inhibitors, including BB-94, prinomastat, GM6001 and DMDP, inhibited the activity of HIV protease (Figure 5B; Supplementary Figure 5D), suggesting that inhibition of HIV infection by MMP inhibitors is independent of HIV protease. Thus, CD62L shedding inhibitors represent a new class of anti-HIV compounds distinct from the existing HIV ART regimen.

### BB-94 inhibited transient upregulation of ADAM10 and 17 expressions during HIV-1 infections

**Figure 6:**
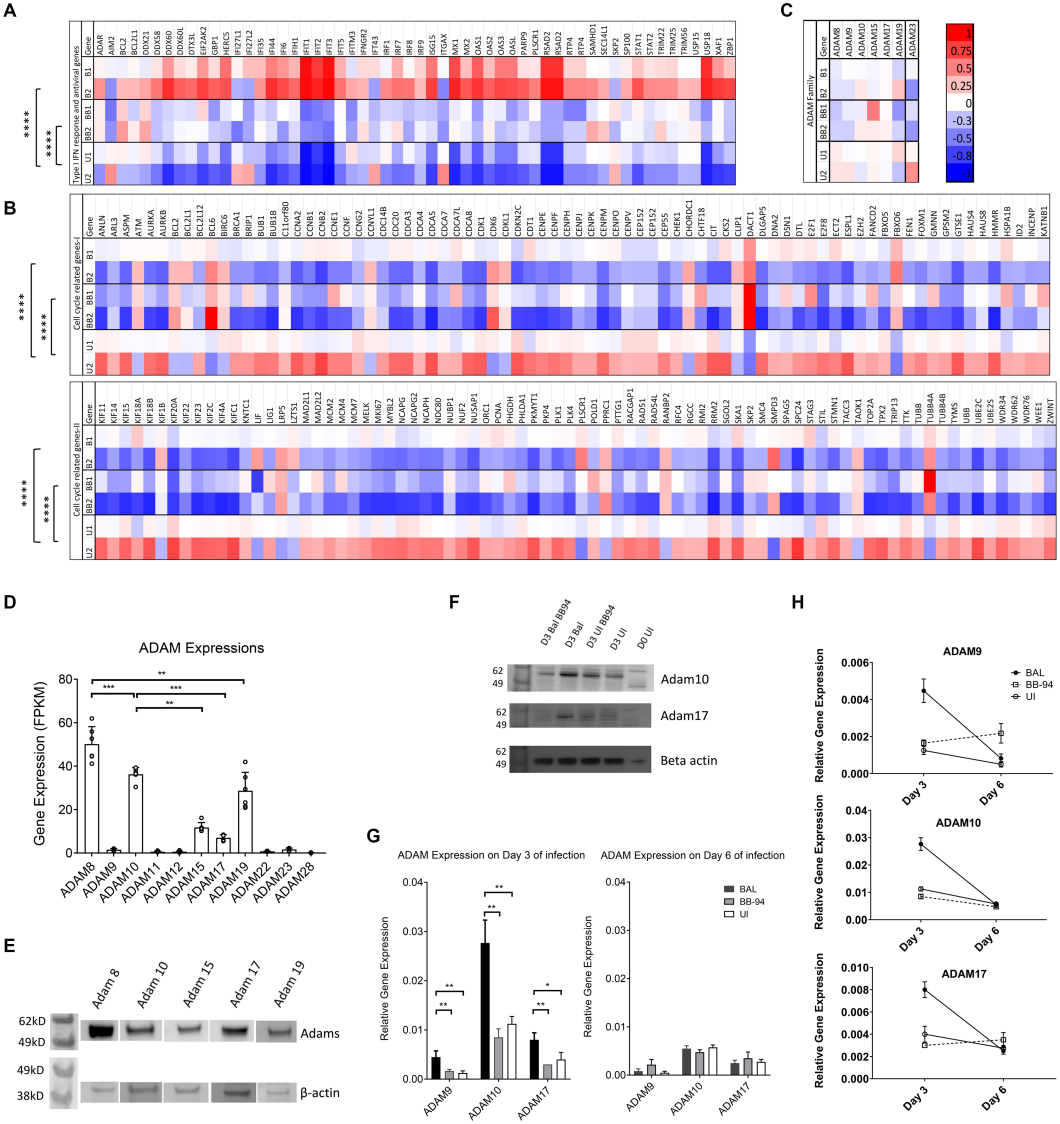
HIV infection transiently induced ADAM expressions. **(A–C)** Heatmap showing differential gene expression analyses of interferon response and antiviral genes **(A)**, cell cycle related genes **(B)**, and various ADAM genes **(C)** from next generation whole cell genome RNA sequencing. B1,B2,U1,U2, BB1,BB2 refer to HIV-1_BAL_ infected (B1,B2), uninfected (U1,U2), and infected samples in the presence of 50 μM BB-94 (BB1, BB2) from cells of two individual healthy donors. The heatmaps are color coded from red (upregulated genes) to blue (down regulated genes). The statistical analyses were done using two-way ANOVA with *p*-values are <0.00001 (****). **(D)** ADAM expression. RNA sequencing data showing the gene expression levels of various ADAMs in CD4 T cells. ADAM8, 9, 10, 15, 17, and 19 expressions are significant by RNA sequencing. The gene expressions are shown as FPKM (expected number of Fragments Per Kilobase of transcript sequence per Millions base pairs sequenced). The overall ADAM expression profiles exhibited similar patterns in PBMC and CD4 T cells. Data include both infected and uninfected samples. **(E)** Western blot analyses of ADAM8, 10, 15, 17 and 19 expressions in uninfected PBMC. **(F)** Wester blot detection of ADAM10 and 17 on day 0 (D0) and 3 (D3) HIV-1 infected versus uninfected samples with and without 50 μM BB-94 treatment. **(G)** RT-PCR analyses on the relative expression of ADAM9, 10 and 17 on day 3 and 6 from uninfected and HIV-1_BAL_ infected PBMC in the presence and absence of BB-94. The expression of all three ADAMs are upregulated in HIV infected cells. **(H)** Relative expressions of ADAM9, 10 and 17 in day 3 and 6 post infection as analyzed by RT-PCR. ADAM expressions were elevated in HIV infected samples on day 3 but returned to the uninfected levels on day 6 of post infection. Statistics are calculated using multiple *t*-tests with *p*-values indicated above their data.*p*-values are *<0.05, **<0.01, ***<0.001.

To characterize the effect of ADAM inhibition on HIV infection, we performed transcriptome analysis by high throughput RNAseq using Illumina platform (Novogene Co. Ltd.) on HIV-1_BAL_ infected with and without BB-94 treatment as well as uninfected CD4 T cells on day 6 post infection (Table 3). Pathway analysis on differentially expressed genes performed using iDEP based on top 400 up- and 400 down-regulated genes showed that HIV-1 infection (samples B1 and B2) significantly up-regulated genes in type I interferon pathway and down-regulated those involved in cell cycle pathways compared to the uninfected (U1 and U2) samples (Sx et al., 2018; Figures 6A,B). This upregulation in type I interferon genes is not observed in the presence of BB-94. However, BB-94 alone does not suppress the transcription of type I interferon pathway genes (Supplementary Figure 6A), suggesting the lack of type I interferon gene upregulation in the BB-94 treated infection is primarily due to the reduction of viral infections in the presence of BB-94. In contrast to genes in type I interferon pathway, HIV-1 infections (B1 and B2) significantly downregulated the cell cycle pathway compared to the uninfected samples (U1 and U2) (Figure 6B). Interestingly, BB-94 alone decreased slightly the expressions of cell cycle genes without HIV-1 infection (Supplementary Figure 6B). Thus, BB-94 does not counter the viral infection in neither interferon nor cell cycle genes, suggesting the antiviral effect of BB-94 is not through regulating the expressions of the two major signaling pathways but rather through its inhibition to the enzymatic activity of metalloproteinases.

**Table 3:** High throughput RNA sequencing statistics.

Samples	Sample type	Total reads	# of total sequences	# of seqs with reads >20	Highest read	Average read	Coding sequences
B1 (BAL)	CD4 T cells	24,214,837	22,660	14,705	444,701	502.7	15,096
B2 (BAL)	CD4 T cells	16,760,043	22,277	13,809	274,368	348.0	15,039
BB1(BB-94)	CD4 T cells	22,492,354	22,675	14,611	350,669	467.0	15,026
BB2(BB-94)	CD4 T cells	19,867,059	22,383	14,085	333,318	412.5	15,043
U1(UI)	CD4 T cells	19,986,304	22,459	14,184	319,908	415.0	15,084
U2(UI)	CD4 T cells	24,709,910	21,692	14,579	463,068	513.1	14,976
UB1	CD4 T cells	15,173,431	25,111	14,304	171,402	258.3	14,797
UB2	CD4 T cells	16,015,876	25,095	14,465	179,586	272.7	14,885
UB3	CD4 T cells	19,832,769	25,568	14,930	218,340	337.7	14,989
U3	CD4 T cells	18,874,099	25,697	14,779	242,850	321.3	14,995
U4	CD4 T cells	16,989,471	24,829	14,514	222,004	289.3	14,826
U5	CD4 T cells	20,959,364	25,637	15,119	260,163	356.8	14,994

BAL, BB-94 and UI are samples from HIV-1_BAL_ infected in the presence of DMSO or BB-94, and uninfected experiments, respectively. Sample 1 and 2 are from donor 1 and 2. The number of total sequences represent all sequences with reads > 0, and they include coding sequences as well as pseudogenes, processed transcripts, Long intergenic non-coding RNAs (LincRNA) and anti-sense sequences. UB1, UB2, UB3 and U3, U4, U5 are BB-94 or DMSO treated uninfected samples from the same donor.

Human CD4 T cells express multiple members of ADAM family genes in addition to ADAM10 and 17 (Figure 6D). The higher expression of ADAM10 than 17 on CD4 T cells suggests ADAM10 contributes more to HIV infection than ADAM17. The expressions of ADAM8, 15 and 19 are also significant on CD4 T cells. The presence of all five ADAMs was confirmed by western blot analysis using antibodies specific to the extracellular domain of individual ADAMs (Figure 6E). Interestingly, while transcriptomic analysis showed no significant differences in ADAM mRNA levels among infected with or without BB-94 and uninfected samples on day 6 of the infection (Figure 6C), western blot analysis showed a marginal increase of ADAM10 and 17 in day 3 infected samples (Figure 6F). To further address if HIV-1 infection resulted in a transient increase in ADAM expressions, we used RT-PCR to quantify ADAM9, 10 and 17 mRNA in infected and uninfected cells from both day 3 and 6 post infection. Indeed, HIV-1_BAL_ infection elevated all three ADAM mRNA levels compared to the uninfected samples only on day 3 but not day 6 of post infections (Figures 6G,H). Their increase was not observed in the presence of BB-94 on both day 3 and 6 of infected samples. Thus, HIV-1 infection induced transient expressions of ADAM10 and 17 in the infected cells.

### BB-94 treatment increased budding virion tethering and suppressed *ex vivo* HIV-1 release

**Figure 7:**
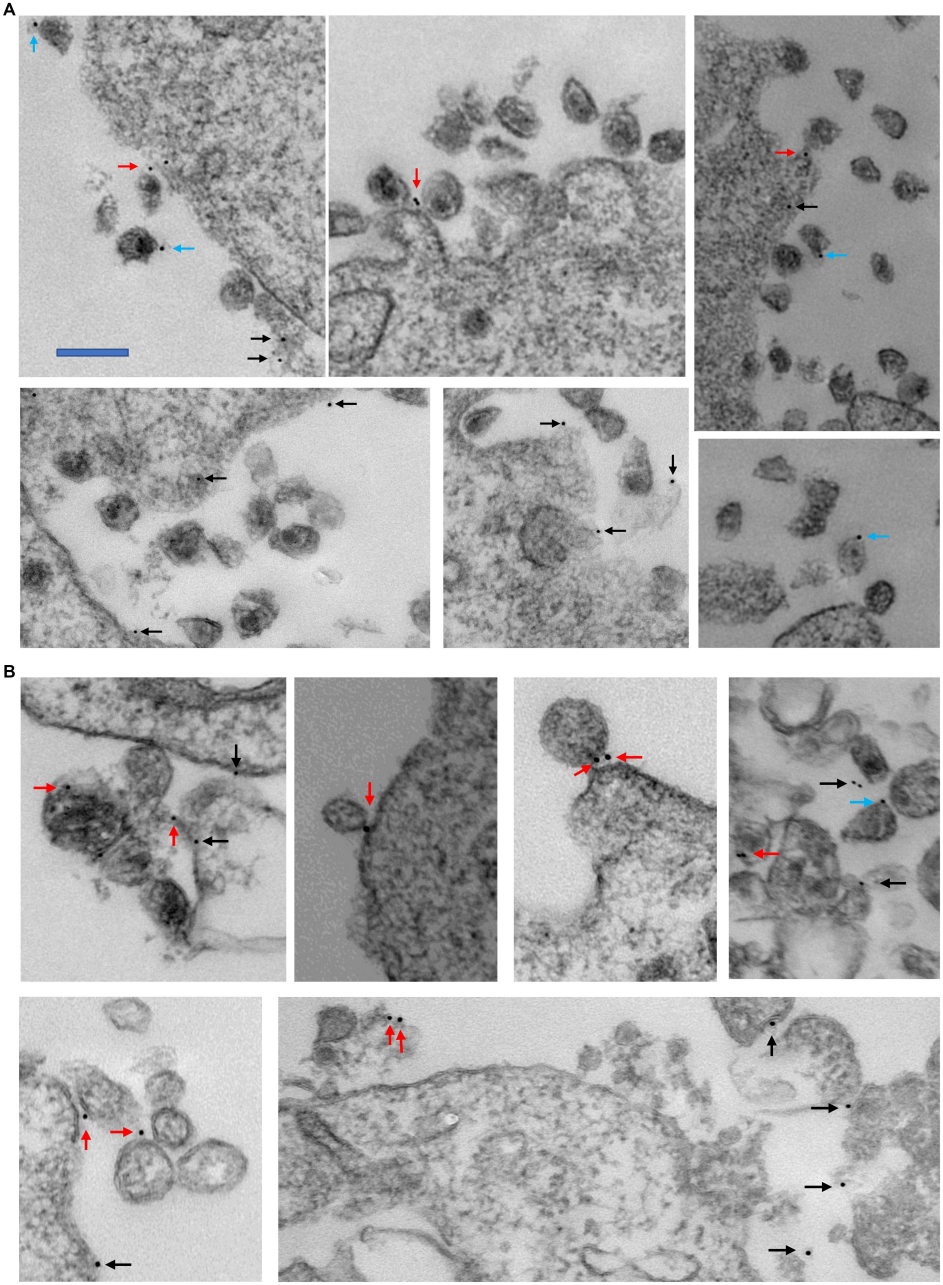
Examples of transmission electron microscopy images showing the staining of 10 nm immunogold labeled CD62L on HIV-1_LAI_ infected PBMC in the absence **(A)** and presence of 50 μM BB-94 **(B)**. The locations of gold particles are indicated by black, red and blue arrows for cell surface non-virion associated CD62L, cell surface virion-tethered CD62L and released virion-bound CD62L, respectively. In the absence of BB-94, ~150 gold particles were found in various TEM images. Majority of them are not associated with virions (presumably bound to cell surface CD62L). Among the 55 virion-associated gold particles, 25 bound to cell surface virions and 31 bound to released virions. In the presence of BB-94, ~250 gold particles were located in TEM images. Only one-third of them are associated with virions. Among them, 63 bound to cell surface virions and 17 bound to released virions. The scalebar is approximately 200 nm.

While CD62L shedding inhibitors suppressed HIV-1 viral infection and deficient budding virions were observed in the presence of BB-94 (Kononchik et al., 2018), it is not clear of the molecular mechanism linking CD62L to HIV-1 release. Recent evidence showed that the viral infection-induced CD62L shedding requires caspase activation and inhibition of caspases impeded the viral release (Segura et al., 2023). CD62L is a C-type lectin receptor capable of binding to HIV-1 envelope glycans (Kononchik et al., 2018). While this glycan binding enhanced viral attachment and entry, it may tether budding virions from release when CD62L shedding is prevented. To visualize CD62L-mediated virion tethering, we labeled X4 tropic HIV-1_LAI_ infected cells in the presence and absence of BB-94 with immunogold particle-labeled anti-CD62L on day 6 of the infection and examined the budding virions using transmission electron microscope (TEM). The CD62L labeling produced fewer than 50 gold-particles/cell to minimize non-specific labeling in the infected and uninfected cells (Figure 7; Supplementary Figure 7). While the majority of immunogold particles labeled cell surface CD62L that were not associated with HIV-1 virions (Supplementary Figure 7C, indicated by black arrows), many immunogold particles (25% of the total gold particles) were observed in contact with HIV-1 virions (Figure 7; Supplementary Figures 7D,E), illustrating the presence of virion-associated CD62L. We further separated the virion-associated immunogold particles into two categories, the ones tethered to budding virions on cell surface (non-cleaved CD62L, indicated by red arrows) and the ones bound to detached virions from cells (cleaved CD62L, indicated by blue arrows) (Supplementary Figures 7D,E). The two categories represent cell surface and shed CD62L. A survey of ~100 TEM images each from the DMSO and BB-94 treated HIV-1 infections showed the fraction of the virion-associated immunogold particles varied between the budding and released virions in DMSO versus BB-94 treated infections. In the absence of BB-94, the fraction of budding virion associated gold-particles (45%, red arrows) is slightly less than that bound to the released virions (55%, blue arrows) (Figure 7A). In the presence of BB-94, however, the fraction of CD62L-tethered virions increased to 79% while those on released virions decreased to 21%. Namely, the ratio of CD62L-tethered budding versus released virions increased from 1:1 to 4:1 in favor of cell surface tethering in the presence of BB-94. Many virions appeared in small aggregates tethered to cell membranes (Figure 7B). The increase in CD62L-tethered budding virions in the presence of BB-94 illustrates the hindrance of cell surface CD62L to the release of budding virions and supports importance of CD62L shedding in HIV-1 viral release.

**Figure 8:**
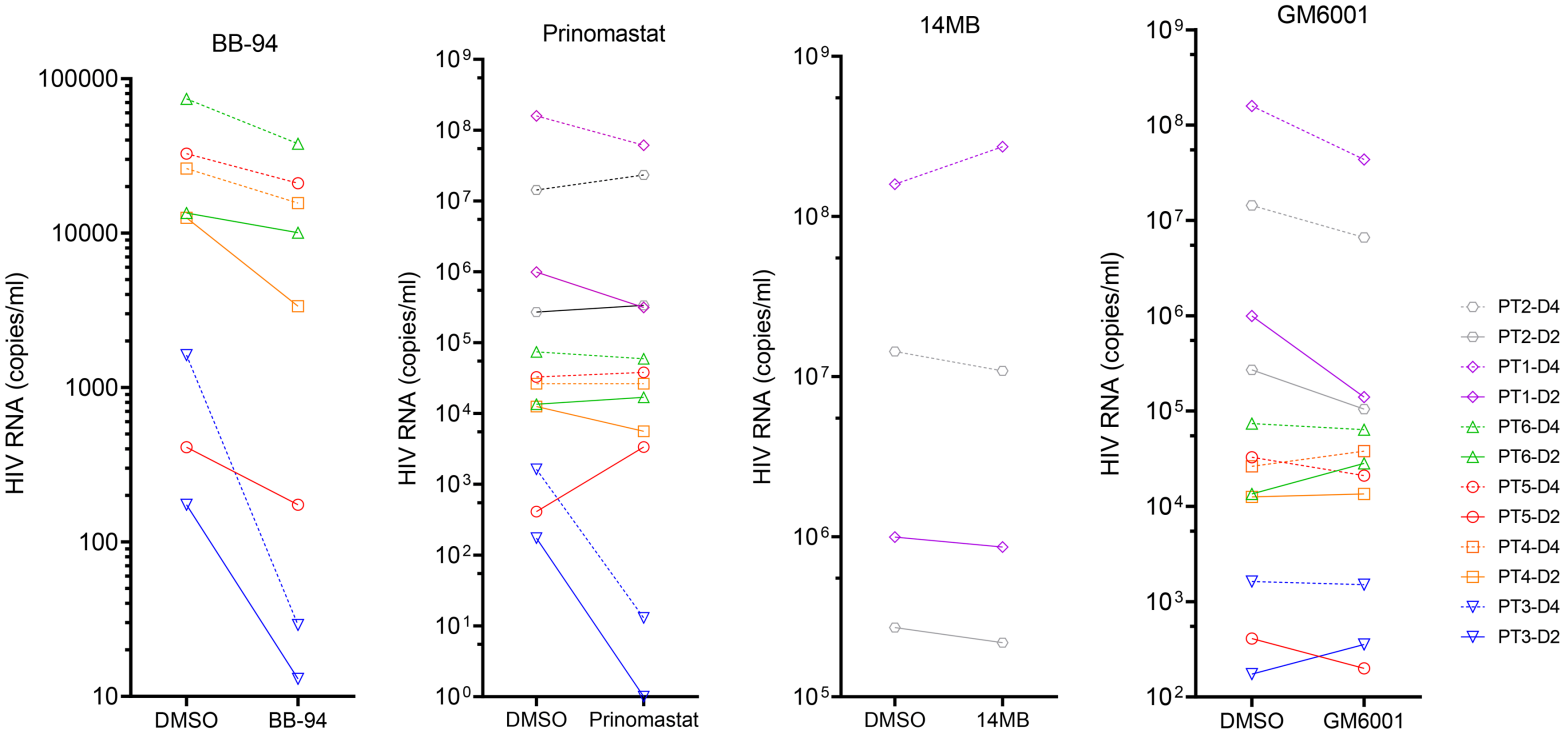
Inhibition of viral release from HIV-1 patient derived CD4 T cells in the presence of 50 μM of indicated compounds or DMSO. The results from day 2 (solid line) and 4 (dash line) samples are displayed on the same graphs organized by the compounds. The statistical analyses were performed based on percentage inhibitions using student’s t-test between DMSO and inhibitor treated experiments (Supplementary Figure 8).

To investigate if CD62L shedding inhibitors affected viral release in clinical specimens, we stimulated CD4 T cells from six HIV-1 infected individuals with anti-CD3 antibody in the presence of BB-94, 14 MB, GM6001 or prinomastat and measured the level of virion-associated HIV RNA released on day 2 and 4 post stimulation. The results showed that BB-94 inhibited the viral release from all individuals’ CD4 T cells (Figure 8), resulting in 2–50 fold reductions in released virion-associated RNA (Supplementary Figure 8). Prinomastat and GM6001 inhibited viral release in a portion of the samples, whereas 14mb fail to inhibit the viral release. It is worth noting that BB-94 was more effective in inhibiting the viral release in individuals with lower viral loads (Figure 8). Together, the results showed that HIV-1 viral release can be inhibited by the most potent metalloprotease inhibitor, suggesting an optimum therapy to inhibit HIV-1 viral release is in conjunction with the existing ART therapy.

## Discussion

**Figure 9:**
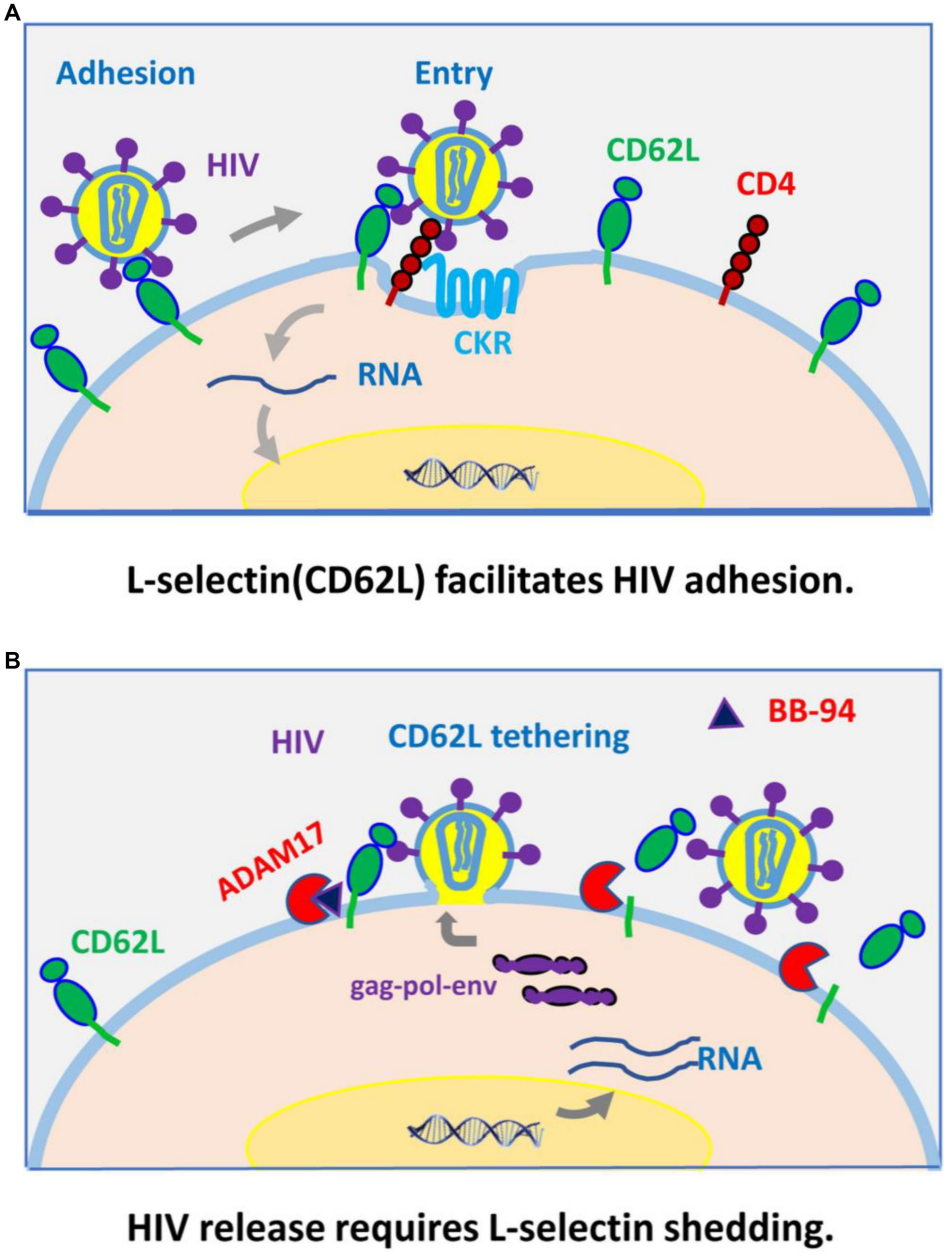
A model for CD62L (L-selectin) as an viral adhesion receptor for HIV-1 entry **(A)**, and its shedding by ADAM metalloproteinases required for HIV-1 budding and release from infected cells **(B)**.

Our recent work showed L-selectin (CD62L) bound HIV-1 envelope glycan and functions as a viral attachment receptor (Kononchik et al., 2018). Upon infection, however, HIV-1 induces the shedding of CD62L resulting in a near complete loss of the receptor in infected T cells. This viral induced shedding of CD62L appears mediated by ADAM metalloproteinases as BB-94 inhibited the receptor shedding. Importantly, inhibition of CD62L shedding also inhibited the viral infection, suggesting the attachment receptor impedes viral release (Figure 9). This paradoxical function of CD62L in HIV-1 infection, facilitating the viral entry but detrimental to viral release, reveals a new therapeutic opportunity to target the viral release using compounds suppressing viral-induced CD62L shedding. In efforts to identify anti-HIV release compounds, we systematically examined the involvement of serine, cysteine, aspartyl-proteases, and Zn-metalloproteinases in CD62L shedding on human lymphocytes using various protease inhibitors. Those compounds inhibited CD62L shedding and suppressed both R5 and X4 tropic HIV-1 infection. They represent a new class of anti-HIV reagents that do not overlap with the current ART compounds. Interestingly, broad specificity inhibitors, such as BB-94, and prinomastat, consistently suppressed the viral infection better than ADAM17 or ADAM 10 specific inhibitors. As CD4 T cells express multiple ADAMs, including ADAM8, 10, 15, 17, and 19, we cannot rule out the involvement of additional ADAMs in HIV infection. Interestingly, the expressions of ADAM10 and 17 were upregulated on day 3 but returned to the uninfected levels on day 6, suggesting that the shedding of CD62L needs to occur prior to HIV-1 release from the infected cells. Mechanistically, inhibition of CD62L shedding by BB-94 resulted in more budding virions tethering on cell surface by CD62L, thus, impeding the viral release (Figure 9).

Conceptually, it is less intuitive to understand why inhibition of adhesion receptor CD62L shedding did not result in an increased viral infection. We think the key resides in which cells shed CD62L and hence are subject of shedding inhibition. Since CD62L downregulation occurred in HIV infected p24+ but not p24- cells as evidenced in an increase of CD4-/CD62L- cells in p24+ but not p24- populations nor in the uninfected samples (Figures 3B,C; Supplementary Figure 4B), thus CD62L shedding is primarily induced by the viral infection. Further, the shedding of CD62L is induced through an HIV Nef-dependent apoptotic caspase activation (Segura et al., 2023). As a result, BB-94 primarily inhibited the shedding of CD62L in infected T cells. Namely, shedding inhibition will likely not affect CD62L expression in uninfected cells and thus would not affect viral entry. It is worth noting that cell-to-cell transfer represents a major route for HIV transmission. While we did not investigate the current compounds in their ability to inhibit HIV infection in a cell-to-cell transfer infection mode, our earlier study showed that BB-94 effectively suppressed HIV cell-to-cell transfer infections (Kononchik et al., 2018). The inhibition of viral release in a cell-to-cell transfer infection is counter intuitive as viral release to the media is not required for virus cell-to-cell transmissions. It is possible that inhibition of CD62L shedding results in not only tethered virions but also intrinsically deficient virions on budding cells, such as the presence of aggregated virions in BB-94 treated samples (Figure 7B).

Finally, the involvement of multiple ADAMs is consistent with our observation that single specificity ADAM inhibitors alone, such as potent ADAM17 inhibitor 14mb or ADAM10 inhibitor GI254023X, were less effective than broad specificity inhibitor BB-94 in suppressing HIV-1 infection. However, due to its broad specificity, BB-94 may have unintended consequences *in vivo* by suppressing metalloproteinases unrelated to HIV release but are involved in normal cellular function (Abel et al., 2004; Wetzel et al., 2017; Schlomann et al., 2019). It is conceivable that the use of a combination of highly potent ADAM isoform specific inhibitors may be more effective in suppressing HIV-1 release than the use of a broad-spectrum inhibitor. With the exception of ADAM10 and 17, however, highly potent isoform specific inhibitors are not available for other ADAMs.

In summary, broad specificity ADAM metalloprotease inhibitors, such as BB-94 and prinomastat, are effective inhibitors of HIV-1 infection through inhibition of CD62L shedding and the viral release. Both ADAM10 and 17 can cleave CD62L for shedding and both appeared to be involved in HIV-1 infection as the single specificity inhibitors generally failed to suppress the viral infection. These CD62L shedding inhibitors constitute a new class of anti-HIV compounds. Mechanistically, inhibition of CD62L shedding resulted in increased receptor tethering of budding virions on CD4 T cells, thus impeding their release. Finally, CD62L shedding inhibitors, in particular BB-94, suppressed HIV-1 release from patient-derived CD4 T cells, demonstrating their potential therapeutic benefit. Importantly, the host regulation to HIV-1 viral release brings novel cellular targets for potential anti-retroviral therapy. As the current ART regiments are insufficient to eradicate the virus, it is intriguing to speculate on the combination of ART with viral release inhibition may bring additional therapeutic benefits since these metalloproteinase inhibitors do not overlap with existing ART compounds in their antiviral activities.

## Materials and methods

### Reagents

Compounds used to inhibit CD62L shedding were purchased from Sigma-Aldrich, Santa Cruz Biotechnology and R&D systems, Inc. HIV protease inhibitors and reverse transcriptase inhibitors were purchased from Sigma-Aldrich, St. Louis, MO. MMP inhibitors were purchased from Sigma-Aldrich, AdooQ BioScience, Irvine, CA or MedKoo Biosciences, Inc., Morrisville, NC. All compounds were used at manufacture recommended effective concentrations or otherwise indicated. Western blot human anti-ADAM antibodies were purchased from R&D systems (R&D systems, Inc., Minneapolis, MN) as follows: anti-ADAM8 (MAB1031 and MAB10311), anti-ADAM10 (MAB1427), anti-ADAM15 (MAB935), anti-ADAM 17 (MAB9301), and anti-ADAM19 (AF5050). Anti-CD3 antibody okt3 was kindly provided by Dr. Gary Gilliland of Janssen Pharmaceuticals, Inc. Human CD62L and TNF-α DuoSet ELISA kits were purchased from R&D systems, Inc.

### Synthesis of compound 14 m and 15

Compounds 14 m and 15 were synthesized as previously described (Li et al., 2010). Both compounds exist as 3-Cl-phenylpyrrolidine and tartrate racemic mixture and were further separated by high performance liquid chromatography (HPLC, Waters Corportion, Milford, MA) prior to linking them to pyrazole. As a result, a total of six conformation variants were obtained, 14ma, 14mb, 15a, 15b, 15c, and 15d. The compounds were not soluble in dimethyl sulfoxide (DMSO) but were dissolved in a 1:1:1 mixture of ethanol:isopropanol:tween 20, referred to as EIT. Among them, 14mb and 15b were the most active racemic form of 14 m and 15. The other conformation variants, including 14ma, 15a, 15c, and 15d did not inhibit the HIV infection.

### Soluble CD62L and TNF-α detection by ELISA

PBMC from de-identified healthy donors were obtained with informed consent from the Department of Transfusion Medicine, National Institutes of Health under Biological reagent Registry RD-14-IX-12 approved by NIH biological safety board. CD8-depleted PBMCs, either stimulated or non-stimulated with 10 μg/mL phytohaemagglutinin (PHA) or anti-CD3 antibody (with or without conjugated beads), were plated at 2×10^6^ cells/ml in 48 well plates in RPMI 1640 + 10%FBS (R10) to a final volume of 1 mL in each well. IL-2 was added at a final concentration of 20 U/mL. CD3 antibody okt3 was used at ~0.2 μg/mL final concentration. Compounds were prepared in appropriate solvents according to the manufacturer’s instructions, diluted to working concentrations using RPMI 1640 + 10%FBS (R10), and added to the cell cultures for 6–24 h for CD62L shedding, and 48 h for TNF-α release. Supernatants were collected and assessed using either the CD62L or TNF-α Duo Set ELISA kit according to manufacturer’s instructions (R&D Systems, Inc.). The viability of cells treated with compounds was assessed by acridine orange / propidiom iodide (AO/PI Logosbio catalog F23001) staining using Luna-FL cell counter following the manufacture’s instruction.^2^

### Enzymatic cleavage assays by ADAM and by HIV proteases

A fluorescence based enzymatic assay was used to measure the cleavage of fluorogenic TNF-α and L-selectin (CD62L) peptides by either recombinant ADAMs or PBMC from healthy donors. The fluorogenic substrate, synthesized by Biomatik^3^, was a Dabcyl (4-(4-dimethylaminophenylazo)benzoyl)- and Edans (5-[(2-aminoethyl)amino]naphthalene-1-sulfonic acid)- conjugated 12 amino acid peptide: Dabcyl-KLDKSFSMIKEG-Edans that corresponds to the extracellular membrane proximal region of CD62L. The fluorescence of the Edans group is quenched by the Dabcyl group in the intact substrate and the cleavage of the CD62L substrate peptide results in a fluorescence emission at 490 nm. The cleavage of fluorogenic TNF-α peptide (ES003, R &D systems, Inc) was detected at emission of 405 nm. The cleavage was measured in a kinetic assay every 3 min for 4 h in black 96-well plates using a Synergy_h1 fluorescent plate reader (BioTek) with 340 nm excitation and 490 nm emission wavelengths. For cleavage using recombinant ADAMs, each enzymatic reaction contained 50–200 ng of recombinant ADAM17 or 10 (R&D systems, Inc) mixed with 5–10 μM fluorogenic TNFα peptide ES003 (R&D systems, Inc) or CD62L peptide with or without indicated inhibitors in 100 μL assay buffer of 25 mM Tris at pH 9.0, 2.5 μM ZnCl2, and 0.005% Brij-35 (w/v). For cell-based cleavage of the fluorescent CD62L-peptide substrate, 300,000 PBMC at 2×10^6^/ml density were mixed with 10 μM fluorescent CD62L-peptide and various inhibitors in a black 96-well plate in 150 μL culture media (RPMI 1640, 10% FBS, 1% penicillin streptomycin). PBMC were stimulated with anti-CD3 antibody for 2–4 days as described above.

HIV-1 protease enzymatic activity was assayed in black 96-well plates at room temperature using a Synergy_h1 fluorescent plate reader with 340 nm excitation and 490 nm emission wavelengths (BioTek). Each enzymatic reaction consisted of a mixture of 100 ng of recombinant HIV-1 protease (Sigma-Aldrich catalog SRP2151), 5 μM concentration of a fluorogenic substrate Arg-Glu(EDANS)-Ser-Gln-Asn-Tyr-Pro-Ile-Val-Gln-Lys(DABCYL)-Arg (Sigma-aldrich catalog H6660), and various concentrations of inhibitor compounds in 100 μL of assay buffer (0.15 M sodium chloride, 1.0 mM EDTA, 1.0 mM DTT, 0.1 M sodium acetate, pH 5.0). The fluorescence of the cleavage reaction was continuously read at every 30 s for a total of 40 min.

### HIV-1 infections

The use of healthy human peripheral blood mononuclear cells (PBMC) is approved by the Department of Transfusion Medicine at the Clinical Center of National Institutes of Health. The use of PBMC from HIV-1 infected individuals for the study was approved under protocol #02-I-0202 by the Institutional Review Board (IRB) of National Institutes of Health with written informed consent from all donors. Peripheral blood mononuclear cells (PBMCs) from anonymous healthy donors were isolated by Ficoll-Paque gradient, and cultured in RPMI 1640 supplemented with 10% FBS, 1%Penicillin/streptomycin, 1% HEPES buffer and 20 U/mL IL-2 (culture media); and stimulated with 0.2 μg/mL anti-CD3 antibody (okt3) for 2 days. CD8^+^ T cells were depleted prior to infection using the Stemcell CD8 positive selection kit according to manufacturer’s instructions. CD8-depleted PBMCs were plated at 2×10^6^ cells/ml in 48-well plates in 1 mL culture media with various inhibitors for 60 min prior to the addition of ~80 TCID_50_ HIV-1_BAL_ or HIV-1_LAI_. Inhibitors were replenished during media change every 3 days. The infections were proceeded with or without inhibitors for 6 days or otherwise specified number of days and then analyzed by FACS staining using fluorescent labeled CD4-BV510, CD62L-APC and CD3-PEC7 antibodies (BD Biosciences). Cells were then washed and permeabilized with CytoFix/CytoPerm (BD Biosciences) solution before intracellular staining for HIV p24 antigen using FITC conjugated anti-p24 antibody clone KC57 (Beckman Coulter, catalog 6604665). All assays were performed with cell viabilities >80%. Replication competent HIV-1 viruses were obtained from the NIH AIDS Reagent Program^4^, and expanded by infecting activated CD8-depleted PBMCs, and TCID_50_ was determined by p24 ELISA.

### ADAM expressions using RT-PCR and western blot analyses

RT-PCR using ADAM9, 10 and 17 primer probes was assayed using TaqMan primer/probe sets from ThermoFisher with a 50 ng cDNA template as described previously (Chun et al., 2013; Kononchik et al., 2018). The following TaqMan 20X Gene Expression Assay probes (Dye: FAM-MGB) were used: ADAM9 (Hs00177638_m1), ADAM10 (Hs00153853_m1), ADAM17 (Hs01041915_m1). Expression Assay standard probes (Dye: VIC-MGB) for β-Actin (Hs01060665_g1) or RNase P/RPPH1 (4403328) were used as gene expression controls. Briefly, HIV infected or uninfected cells from triplicate experiments were washed 3x in PBS and lysed for RNA isolation using RNeasy mini kit (QIAGEN corp). The corresponding cDNA samples were prepared using either TaqMan microRNA reverse transcription kit or Invitrogen High-Capacity cDNA reverse transcription kit (Thermo Fisher). Samples were run in duplicate across triplicate experiments on an ABI 7300 Real-Time PCR System (Applied Biosystems) with a threshold of 0.2. Relative gene expression was calculated using the 2^-ΔCt^ method and normalized to β-Actin or RNaseP as the endogenous controls. All statistical analyses were carried out using the software Prism 8 (GraphPad Software, Inc). Western blot analyses were used to detect the mature active form of ADAMs (Pereira Vatanabe et al., 2021). In brief, total protein was extracted using RIPA lysis buffer (catalog 89900, Thermo USA) either from 60×10^6^ million PBMC for the expression of ADAM8, 10, 15, 17 and 19, or from 20 ×10^6^ HIV-1_BAL_ infected or uninfected PBMC for ADAM10 and 17 on day 3 of post infection. SDS samples of protein extracts were electrophoresed, transferred to PVDF (IB34002, Thermo, USA) membranes, blocked with 5% non-fat dry milk (catalog 1,706,404, BioRad, USA) containing 0.1% Tween20 (catalog P1379, Sigma, USA) for 2 h at RT, and incubated overnight at 4°C with antibodies against ADAM8 (1:1000 dilution, catalog MAB10311, R&D systems), ADAM10 (1:1000 dilution, catalog ab1997, Abcam, USA), ADAM15 (1:1000 dilution, catalog MAB935, R&D systems), ADAM17 (1:1000 dilution, catalog PA5-11572, Invitrogen, USA), ADAM19 (1:1000 dilution, catalog ab191457, Abcam), or beta-Actin (1:1000 dilution, catalog MA5-15739, Invitrogen, USA), respectively. The membranes were washed three times and incubated for 1 h at room temperature with the appropriate HRP-conjugated secondary antibodies: rabbit anti-mouse IgG (Abcam catalog 6728), goat anti-rabbit IgG heavy chain (Invitrogen catalog A271036). Detection of protein expression levels by enzyme-linked chemiluminescence (catalog 34580, Thermo, USA) was performed according to the manufacturer’s protocol.

### RNA sequencing experiments

CD4+ T cells were enriched from PBMC using EasySep^™^ human CD4 T cell enrichment kit (STEMCELL Technologies Inc., MA 02142). To prepare samples for RNA sequencing, 5-10×10^6^ PBMC or CD4+ T cells from either infected or uninfected samples were lysed for RNA isolation using RNeasy mini kit from QIAGEN. Samples of 2–10 μg RNA with concentration > 25 ng/μl and OD260/280 > 2.0 were used for cDNA library preparation of 250–300 base pair fragments in length, and high throughput sequencing using Illumina Miseq platform by Novogene (Novogene Corp Inc., CA 91914). A total of ~20×10^6^ reads were recorded for each sample (Table 3). The mapping and assembly of raw sequencing reads, statistical and preliminary bioinformatic analyses were performed by Novogene. Subsequent gene expression analyses were performed based on expected Fragments Per Kilobase of transcript sequence per Millions base pairs sequenced (FPKM) using sequences of 20 reads or higher. All differential gene expression analyses were done between infected samples in the presence or absence of treatment and their donor matched uninfected controls. Cellular pathway analyses in response to HIV-1 infection in the presence and absence of BB-94 treatment were analyzed by inputting the top 400 upregulated and 400 down regulated genes from the RNAseq data to a bioinformatics software iDEP.96 (bioinformatics.sdstate.edu/idep/) using generally applicable gene set enrichment (GAGE) method (Luo et al., 2009; Sx et al., 2018). The RNAseq data is available in GEO database under accession number GSE218175.

### Transmission electron microscopy (TEM)

CD8-depleted PBMC were infected with HIV-1_LAI_ in the presence and absence of 100 μM BB-94 as described in the methods. For immunogold labeling, ~10^6^ cells were washed with 2 mL PBS, gently centrifuged at 250 *g* for 5 min, resuspended in 100 μL of labeling buffer (PBS with 1% BSA), and incubated with 10 μg of mouse anti-human CD62L (clone FMC46, Thermofisher) for 1 h on ice. After removal of the primary antibody and wash, cells were incubated with 10 μg of secondary goat anti-mouse IgG H&L conjugated to 10 nm gold nanoparticles (Electron Microscopy Sciences) for 1 h on ice, then washed and fixed in 2.5% glutaraldehyde with 0.1 M sodium *cacodylate* at pH 7.4. Specimens for transmission electron microscopy (TEM) were fixed with 2.5% glutaraldehyde in 0.1 M Sorenson’s buffer. Samples were post-fixed 1 h with 0.5% osmium tetroxide/0.8% potassium ferricyanide, 1 h with 1% tannic acid and overnight with 1% uranyl acetate at 4°C. Samples were dehydrated with a graded ethanol series and embedded in Spurr’s resin. Thin sections were cut with a Leica UCT ultramicrotome (Vienna, Austria) stained with 1% uranyl acetate and Reynold’s lead citrate prior to viewing at 80 kV on a Hitachi 7500 transmission electron microscope (Hitachi-High Technologies, Tokyo, Japan). Approximately 100 images were acquired with an AMT digital camera system (AMT, Chazy, NY) from each sample on sections containing HIV virions. HIV virions are identified as ~100 nm sized particles with visible membrane enclosed capsid. The immunogold labeling resulted in less than 50 gold-particles/cell to minimize non-specific labeling.

### *Ex vivo* HIV-1 release assay

PBMCs were collected from the study subjects in accordance with a clinical protocol approved by the Institutional Review Board of the National Institute of Allergy and Infectious Diseases, National Institutes of Health under protocol #02-I-0202. CD4^+^ T cells were isolated from the PBMCs using a human CD4 T cell separation system (StemCell Technologies, Catalog 19052) and were cultured with medium alone or with plate-bound anti-CD3 and soluble anti-CD28 antibody in the absence or presence of 50 μM of indicated compounds for 48 h. The copy number of cell-free viron-associated HIV RNA in the culture supernatants was quantified using the Cobas Ampliprep/Cobas Taqman HIV-1 Test, Version 2.0 (Roche Diagnostics) with droplet digital PCR (ddPCR, BIO-RAD) on day 2 and 4 of stimulation.

### Statistical analyses

All experiments have been repeated at least two times and the results presented here are representative of the duplicates without data rejection. All statistics are calculated using multiple *t*-tests with *p*-values either indicated above their data or expressed as * = <0.05, ** = <0.005, *** = <0.0005, **** = <0.00005. Normalized inhibition data in Figure 6 are fitted with a non-linear regression model using GraphPad Prism 8.0.1.

## Data availability statement

The RNAseq data presented in the study are deposited in NCBI GEO repository, accession number GSE218175.

## Ethics statement

The studies involving humans were approved by NIAID Institutional review board protocol #02-I-0202. The studies were conducted in accordance with the local legislation and institutional requirements. The participants provided their written informed consent to participate in this study.

## Author contributions

JI: Data curation, Formal analysis, Investigation, Methodology, Writing – review & editing. JS: Data curation, Formal analysis, Investigation, Methodology, Writing – review & editing. GS: Data curation, Methodology, Resources, Writing – review & editing. JB: Data curation, Formal analysis, Investigation, Methodology, Writing – review & editing. GR: Data curation, Formal analysis, Methodology, Writing – review & editing. TS: Data curation, Formal analysis, Methodology, Writing – review & editing. RW: Data curation, Formal analysis, Methodology, Writing – review & editing. XJ: Methodology, Resources, Writing – review & editing. EF: Data curation, Formal analysis, Investigation, Methodology, Writing – review & editing. SM: Methodology, Resources, Writing – review & editing. T-WC: Data curation, Formal analysis, Investigation, Methodology, Writing – review & editing. PS: Conceptualization, Funding acquisition, Investigation, Supervision, Writing – original draft, Writing – review & editing.

## Footnotes

^1^ https://www.who.int/news-room/fact-sheets/detail/hiv-aids

^2^ Logosbio.com

^3^ www.biomatik.com

^4^ https://www.hivreagentprogram.org/
