# Supplementary material for: Inhibition of HIV-1 release by ADAM metalloproteinase inhibitors
Source: Front Microbiol. 2024 Mar 20;15:1385775. doi: 10.3389/fmicb.2024.1385775 (PMC10987949; doi:10.3389/fmicb.2024.1385775)
Supplement: Supplementary file 1 [file Data_Sheet_1.PDF]

Supplemental Figures

Supplemental Figure 1

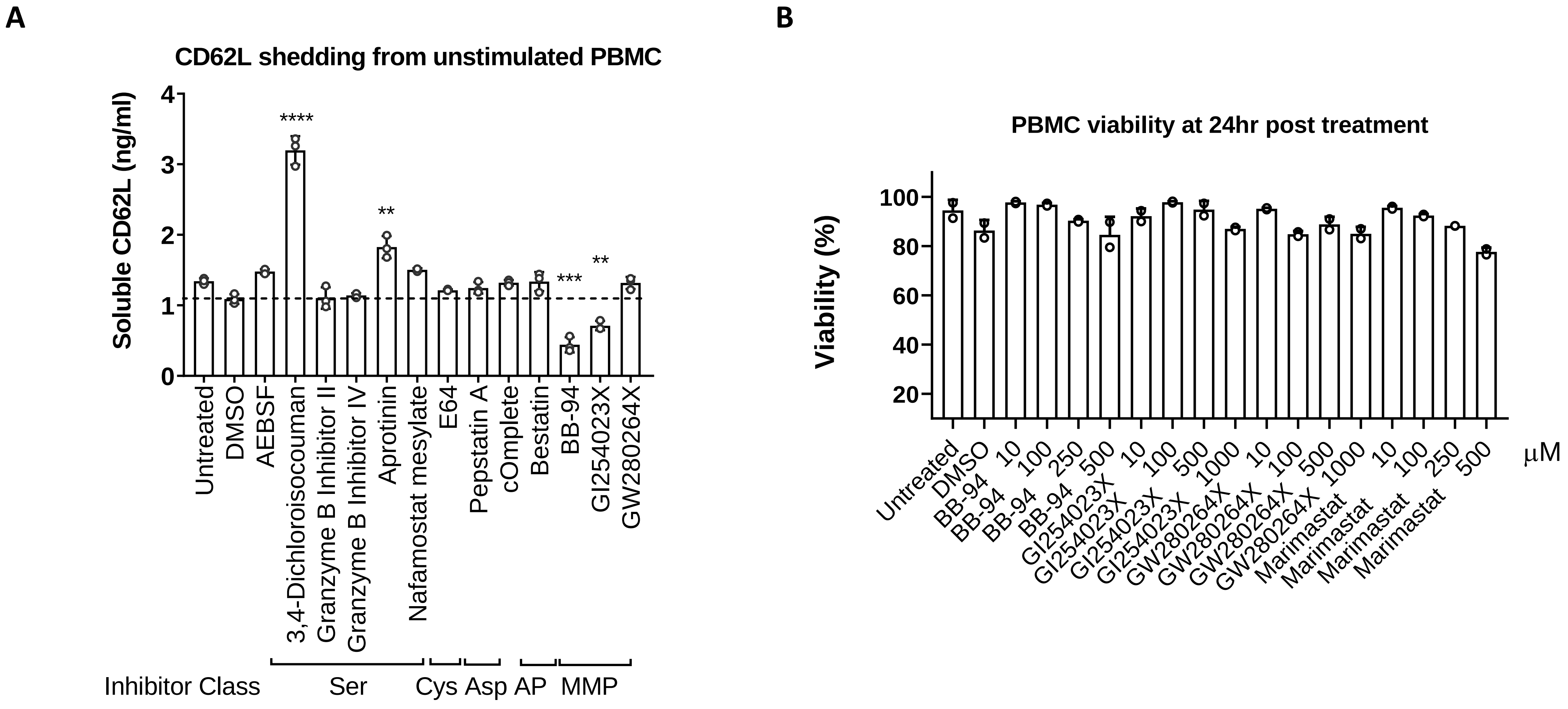

Supplemental Figure 1. CD62L shedding from unstimulated PBMC. A) Soluble CD62L in the supernatant of unstimulated PBMC was measured By ELISA (R&D systems, Inc) after 24 hour treatment in the presence of 10 μM of serine (Ser), cysteine (Cys) , aspatyl (Asp), aminopeptidase (AP) protease inhibitors, respectively, or their untreated controls. Statistics were calculated using unpaired parametric student t-test between individual treatment and DMSO control. P-values are ** <0.005, ***<0.001, ****< 0.0001. B) Viability of PBMC treated with 10-500 μM of MMP inhibitors for 24hours. No significant decrease in the viability observed in these treatment.

Supplemental Figure 2

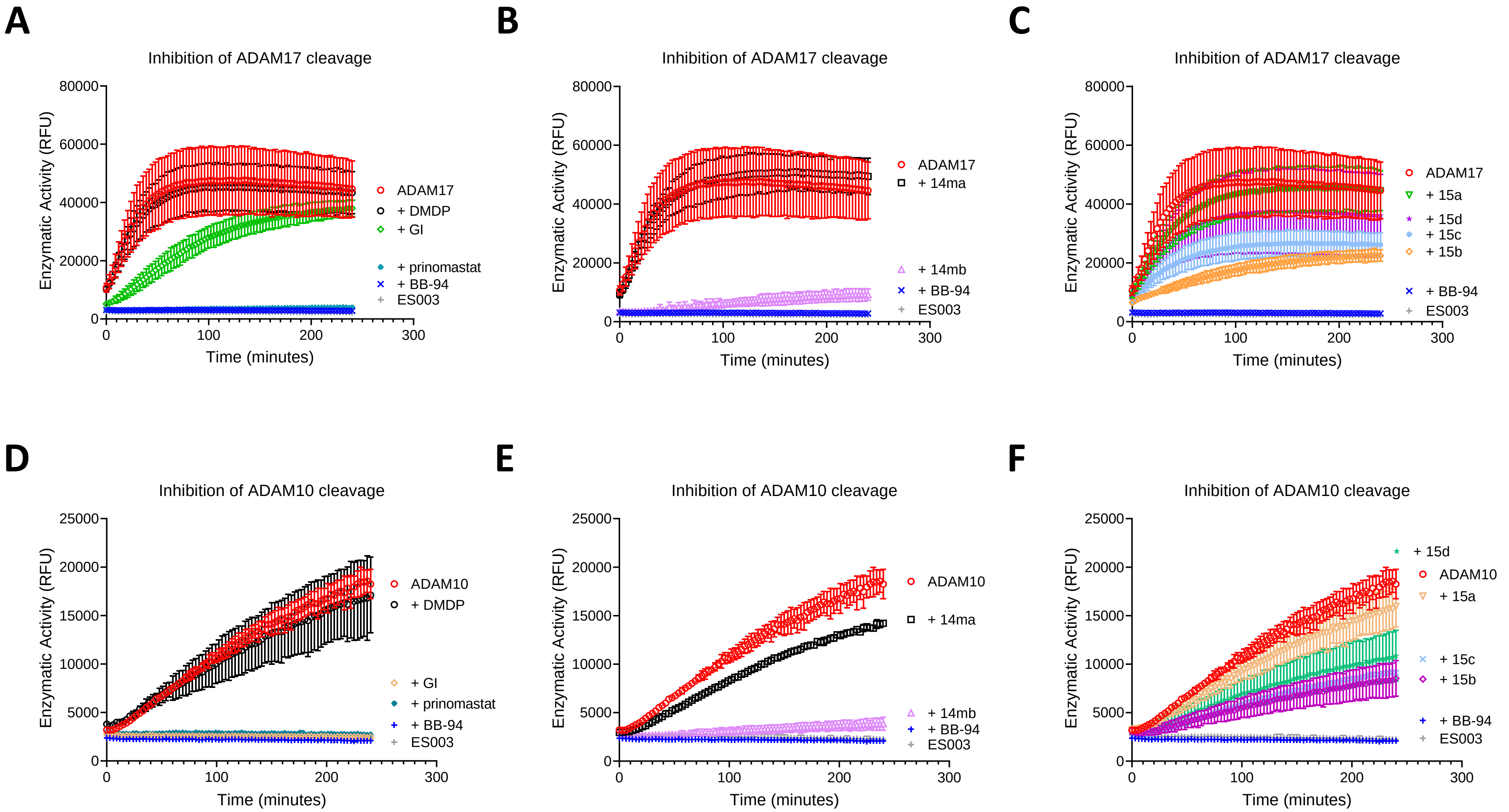

Supplemental Figure 2. Inhibition of ADAM17 (A-C) or ADAM10 (D-F) cleavage of a fluorogenic TNF-α peptide substrate ES003 in the presence of 2μM indicated compounds. The cleavage of fluorogenic TNF-α peptide (ES003, R &D systems, Inc) was detected at emission of 405nm in a kinetic assay using a 96-well plate with data recorded every 3 minutes for 4 hours using a Synergy_h1 fluorescent plate reader. Each enzymatic reaction contained 50-200 ng of recombinant ADAM17 or 10 (R & D systems, Inc) mixed with 5-10 μM fluorogenic TNFα peptide ES003 (R&D systems, Inc) with or without indicated inhibitors in 100 μL assay buffer of 25 mM Tris at pH 9.0, 2.5 μM ZnCl2, and 0.05% Brij-35 (w/v). Figure 2B and 2C bar diagrams are generated using the data points at 4 hour time point of the enzymatic cleavages.

Supplemental Figure 3

A

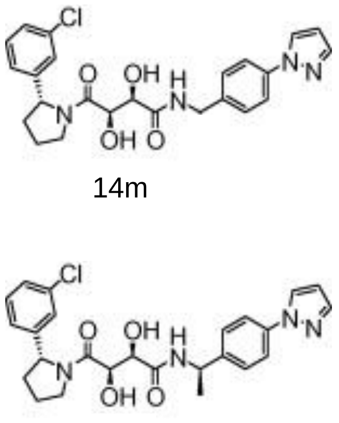

14m

15

B

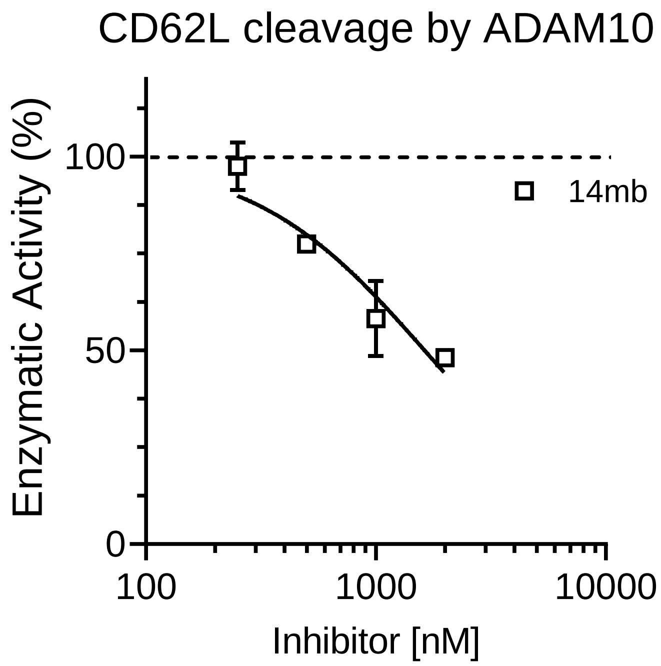

C

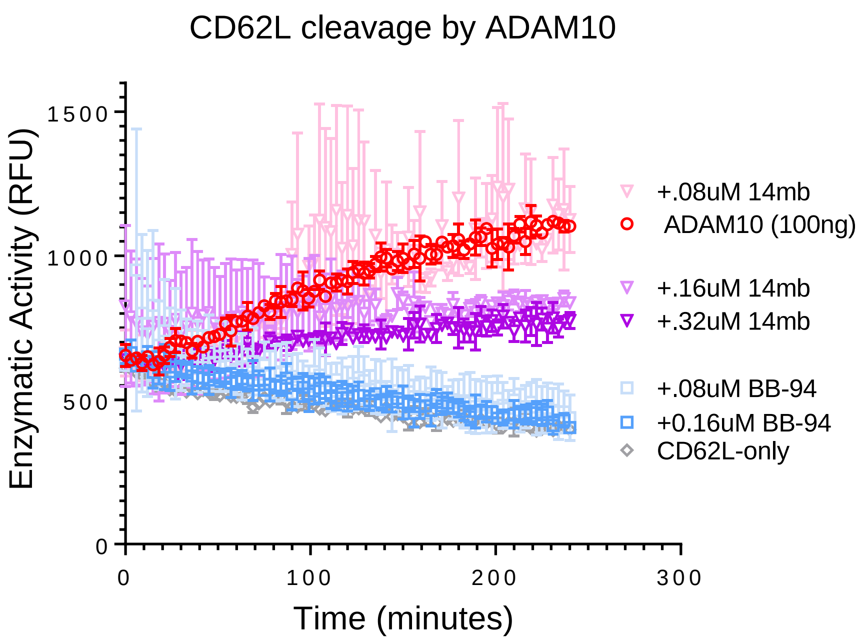

Supplemental Figure 3. Inhibition of ADAM10 enzymatic activity by 14mb. A) chemical structure of 3-Cl-phenylpyrrolidine tartrate diamide analogs 14m and 15. B)Inhibition of recombinant ADAM10 cleavage of a fluorogenic CD62L peptide substrate in the presence of titrating concentrations of 14mb. The concentration-dependent enzymatic activities are fitted with non-linear regression of normalized inhibition response model. C). Inhibition of ADAM10 enzymatic cleavage of fluorogenic CD62L peptide by various concentrations of 14mb and BB-94.

Supplemental Figure 4

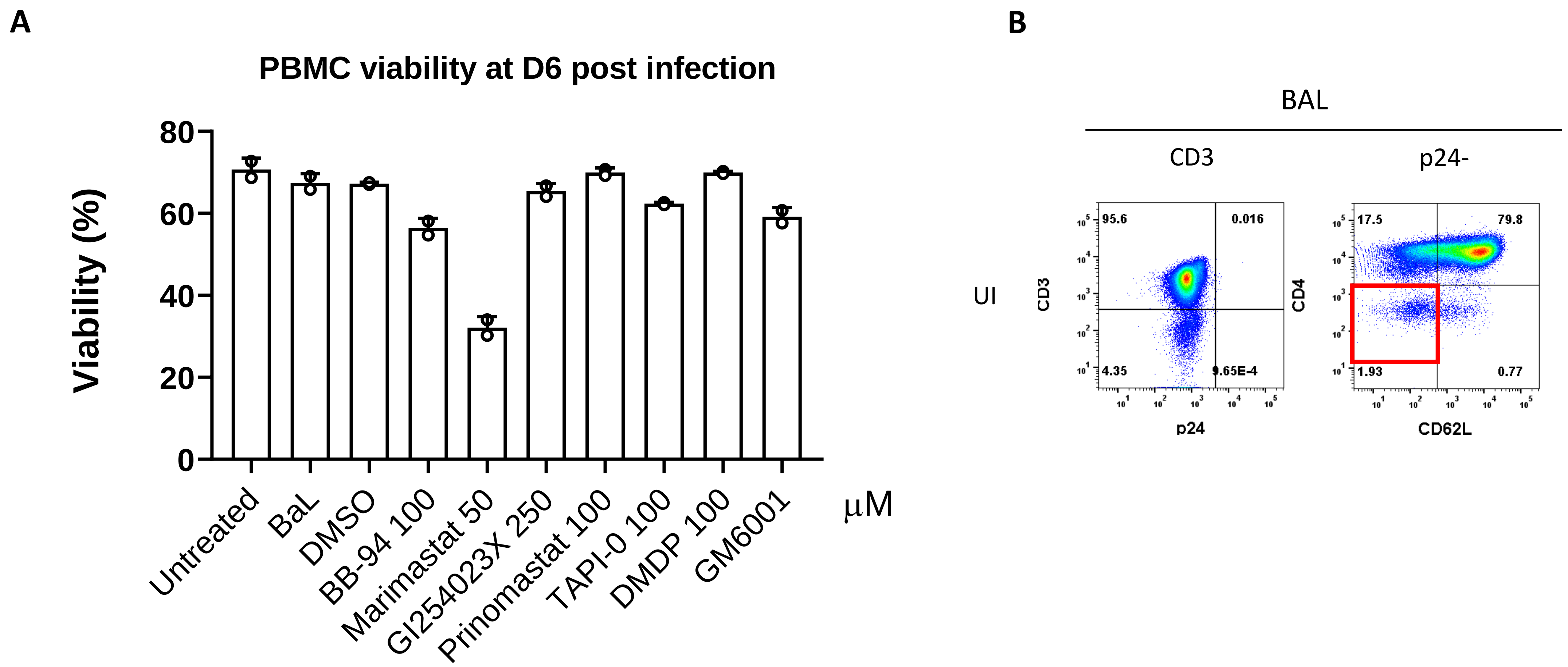

Supplemental Figure 4. Viability and control experiments. A)Viability of PBMC at day 6 post infection with HIV-1 BAL. Marimastat at 50uM resulted in significant loss in cell viability. B) FACS analysis of the uninfected samples in HIV-1 BAL infection experiment. The CD62L-/CD4- population is highlighted in red region.

Supplemental Figure 4

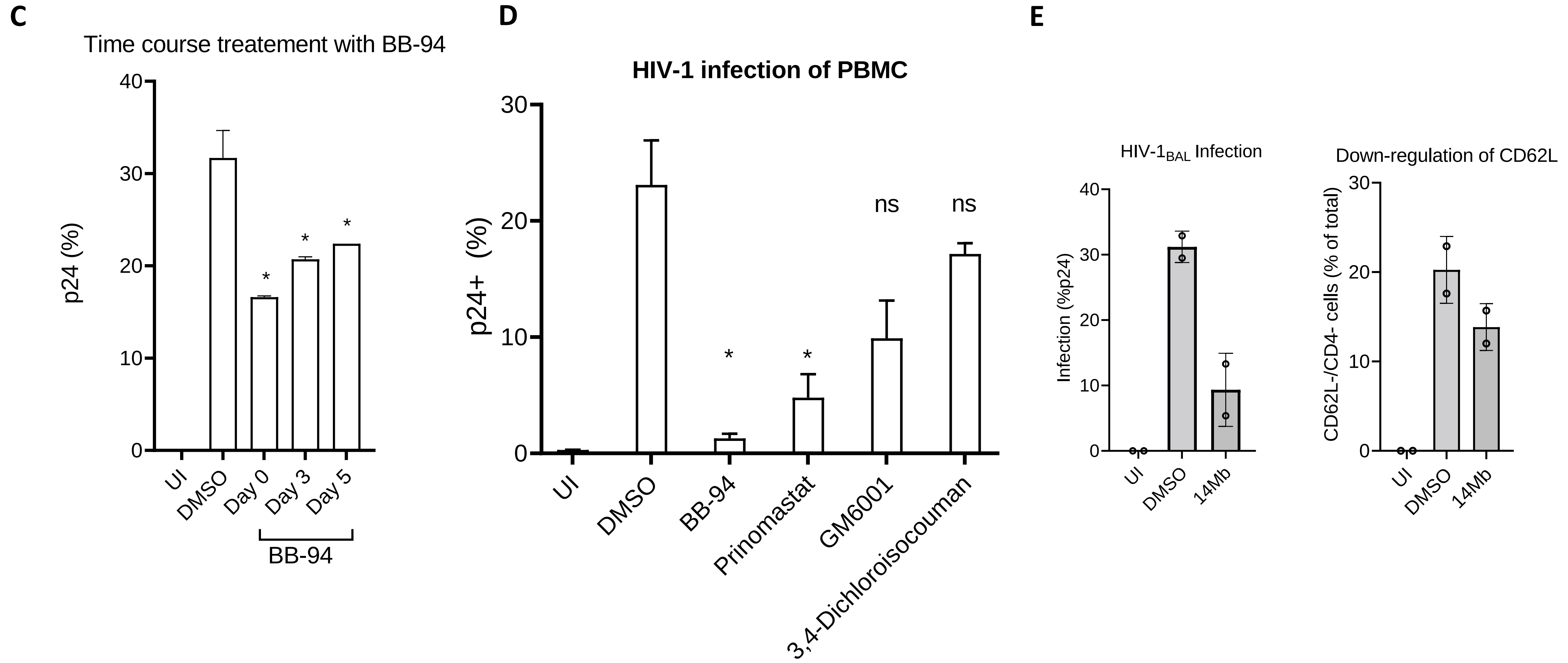

Supplemental Figure 4. C) Addition of 100μM BB-94 on day 0, 3, and 5 post infection. D) HIV-1_{BAL} infection of PBMC at day 6 post infection in the presence and absence of 100 μM indicated compounds. The cells were intracellularly stained with anti-p24 antibody, and the percentage of p24+ cells were shown. The p-values are calculated between DMSO and compound treated infections using student-t test. E) Inhibition of 14Mb to HIV-1 infection (left) and the down-regulation of CD62L expression in infected cells.

Supplemental Figure 5

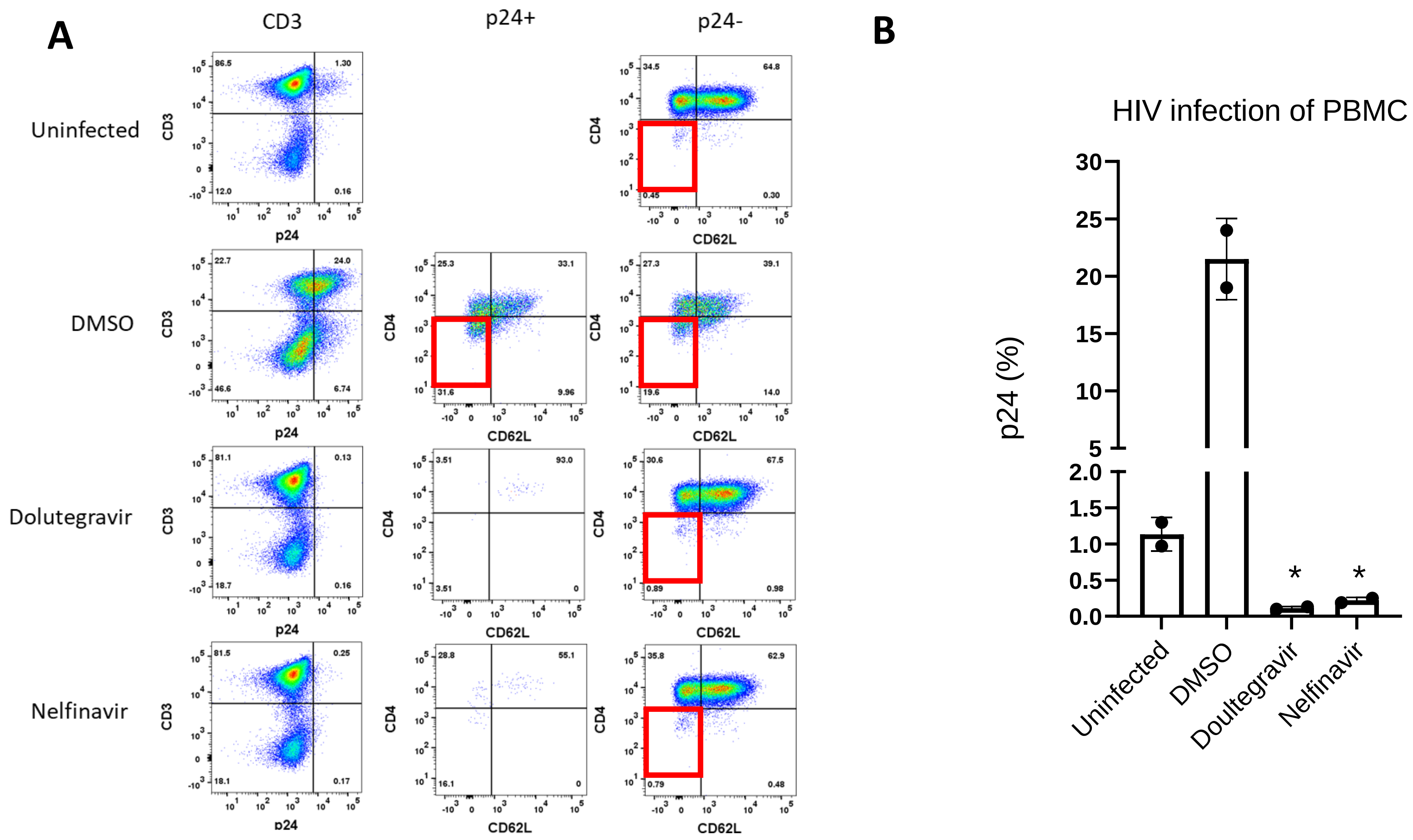

Supplemental Figure 5. HIV-1_{BAL} infection of PBMC. A) FACS analyses of day 6 infections in the presence of 0.5μM compounds. The cells were gated on CD3 and intracellular p24 staining (left column). The CD3+/p24+ (middle column) and CD3+/p24- (right column) cells were further gated by CD4 and CD62L antibodies. Cells downregulated CD4 and CD62L are in the red box region. B) Bar diagram of panel A. The percentage of p24+ cells stained by the anti-p24 represent productive infected cells as no significant staining were detected with the two antiviral compounds. The p-values are calculated between DMSO and compound treated infections using student-t test.

Supplemental Figure 5

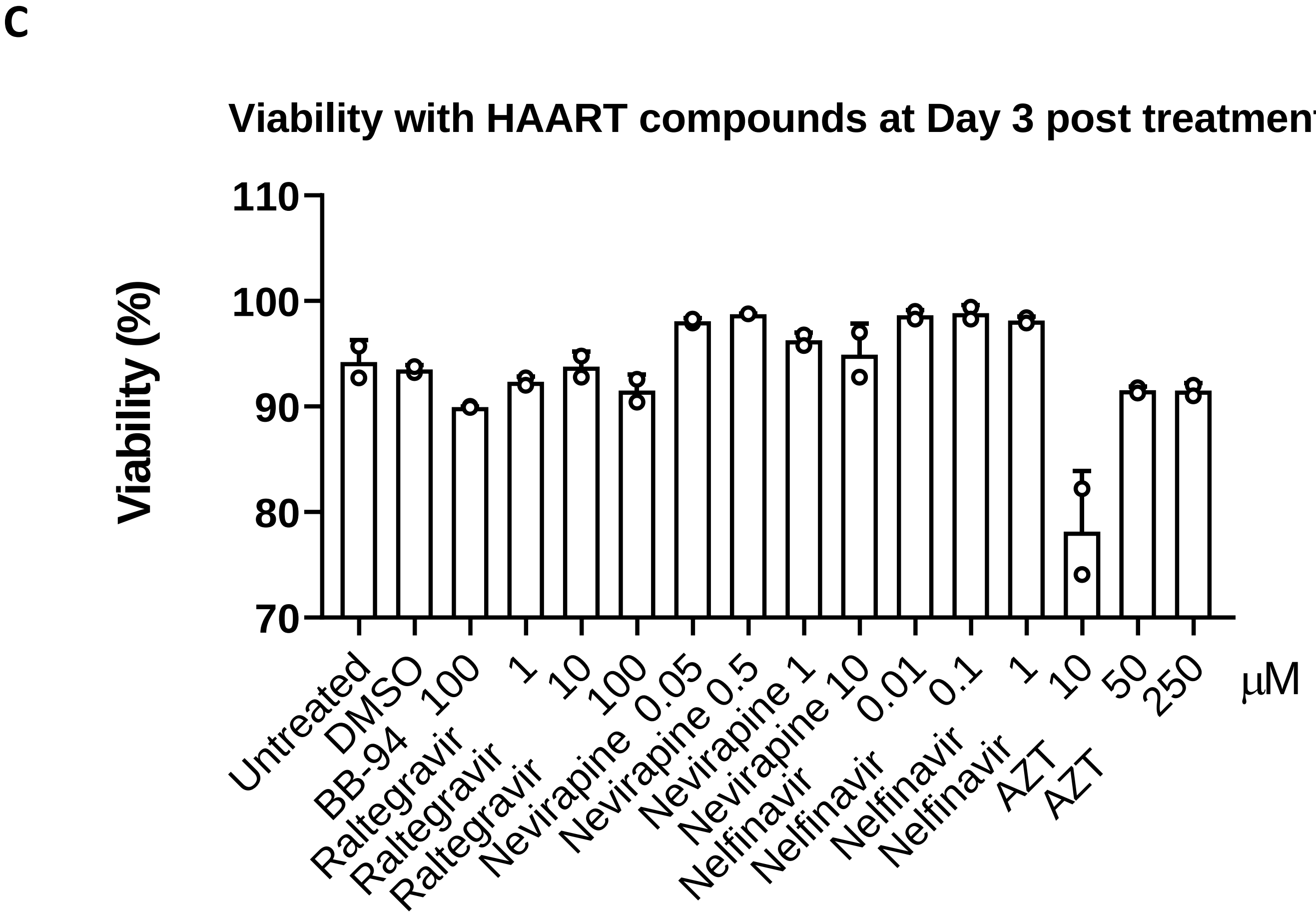

Supplemental Figure 5C. Viability of PBMC treated with concentration dependent HAART compounds at day 3. Nelfinavir treatment at 10uM resulted in significant reduced cell viability.

D

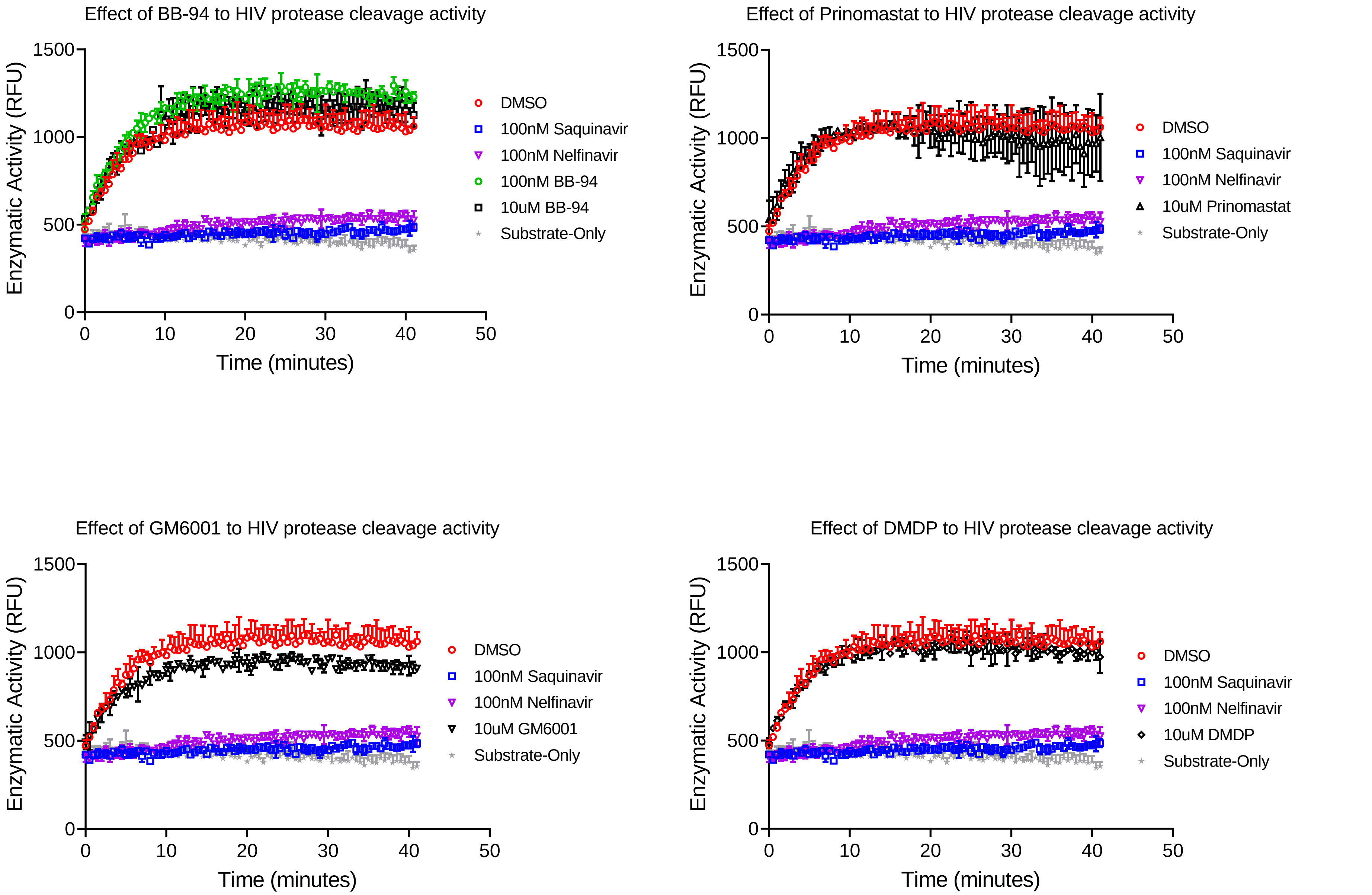

Supplemental Figure 5D. Inhibition of HIV protease cleavage of substrate by MMP inhibitors.

Supplemental Figure 6

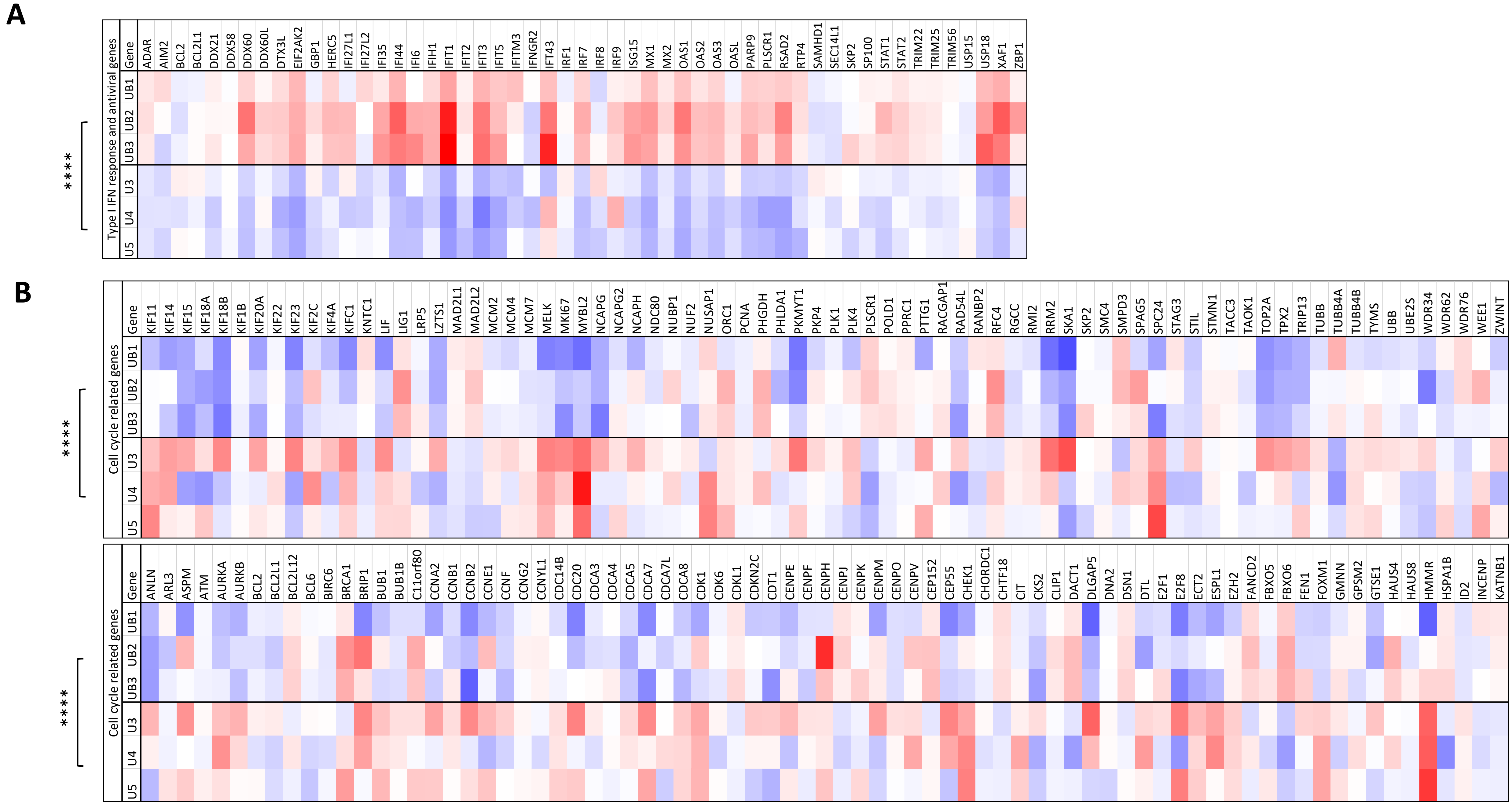

Supplemental Figure 6. Effect of BB-94 treatment to interferon and cell cycle gene expressions. A) Heatmap showing differential gene expression analyses of interferon response and antiviral genes (A) and cell cycle related genes (B) from next generation whole cell genome RNA sequencing. UB1,UB2,UB3 and U3,U4, U5 are BB-94 or DMSO treated uninfected CD4 T cells in triplicates, respectively. The heatmaps are color coded from red (upregulated genes) to blue (down regulated genes) in the same range as Figure 6. Statistical analyses are performed using two-way ANOVA with p-values are <0.00001 (****).

Supplemental Figure 7

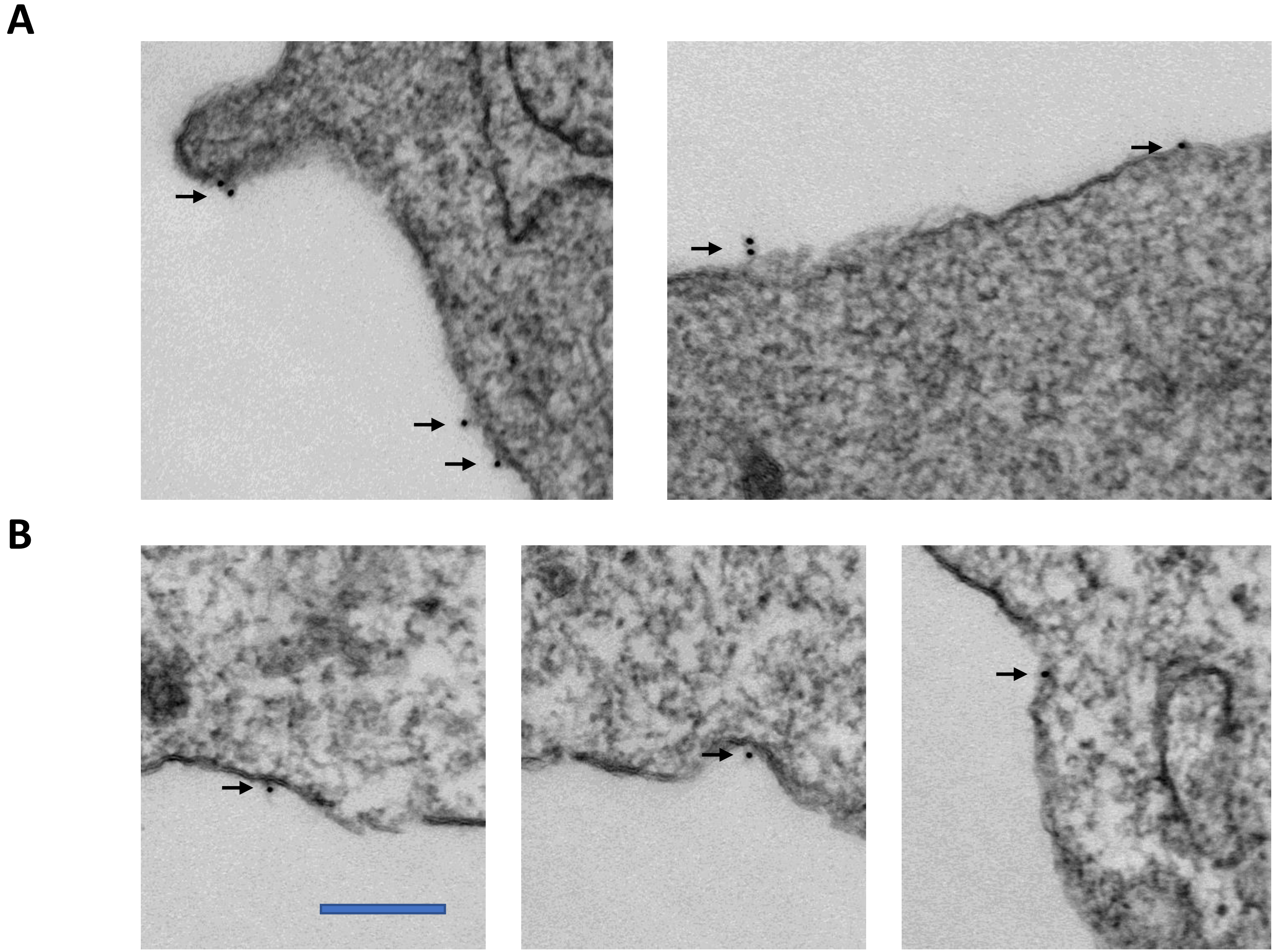

Supplemental Figure 7

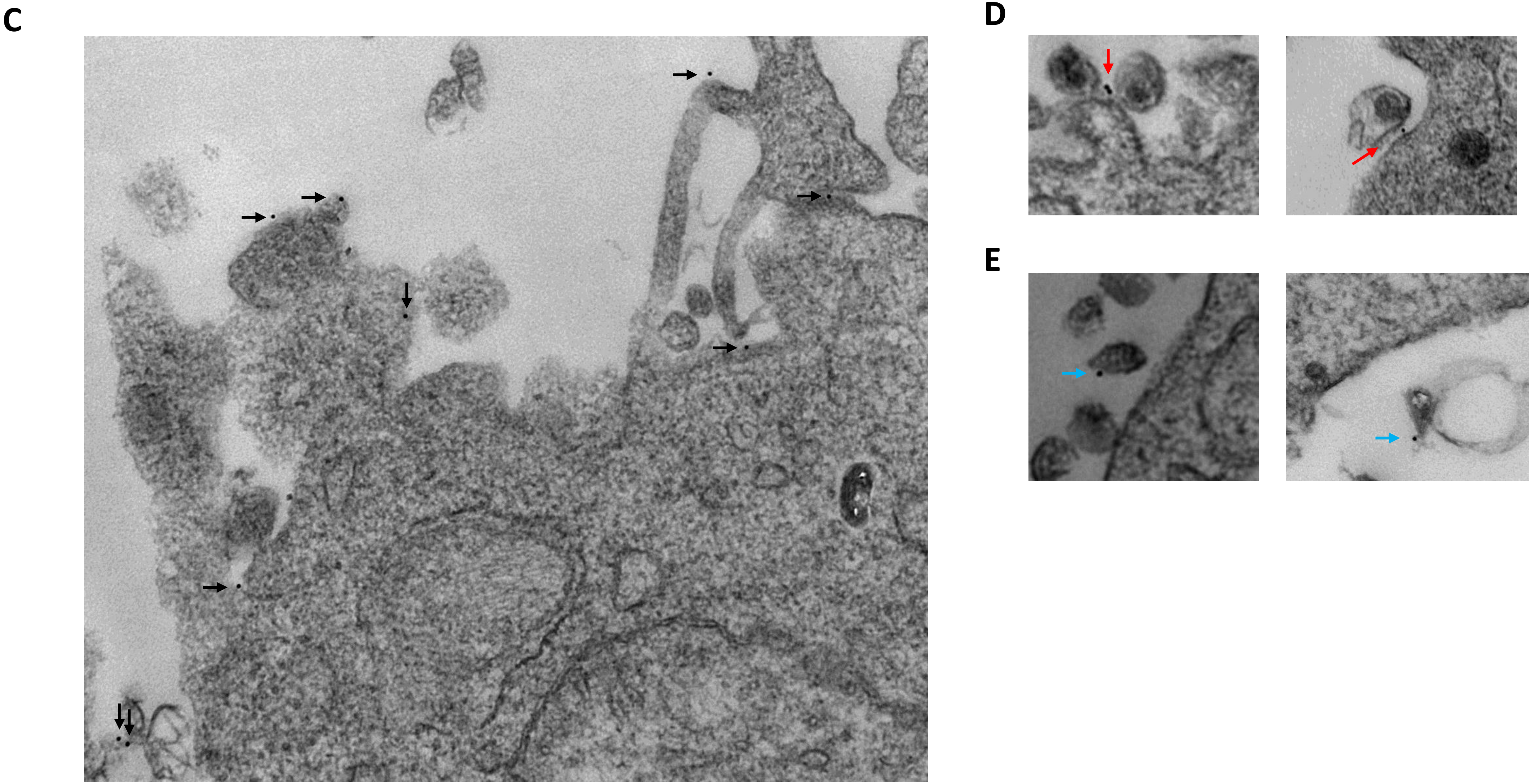

F

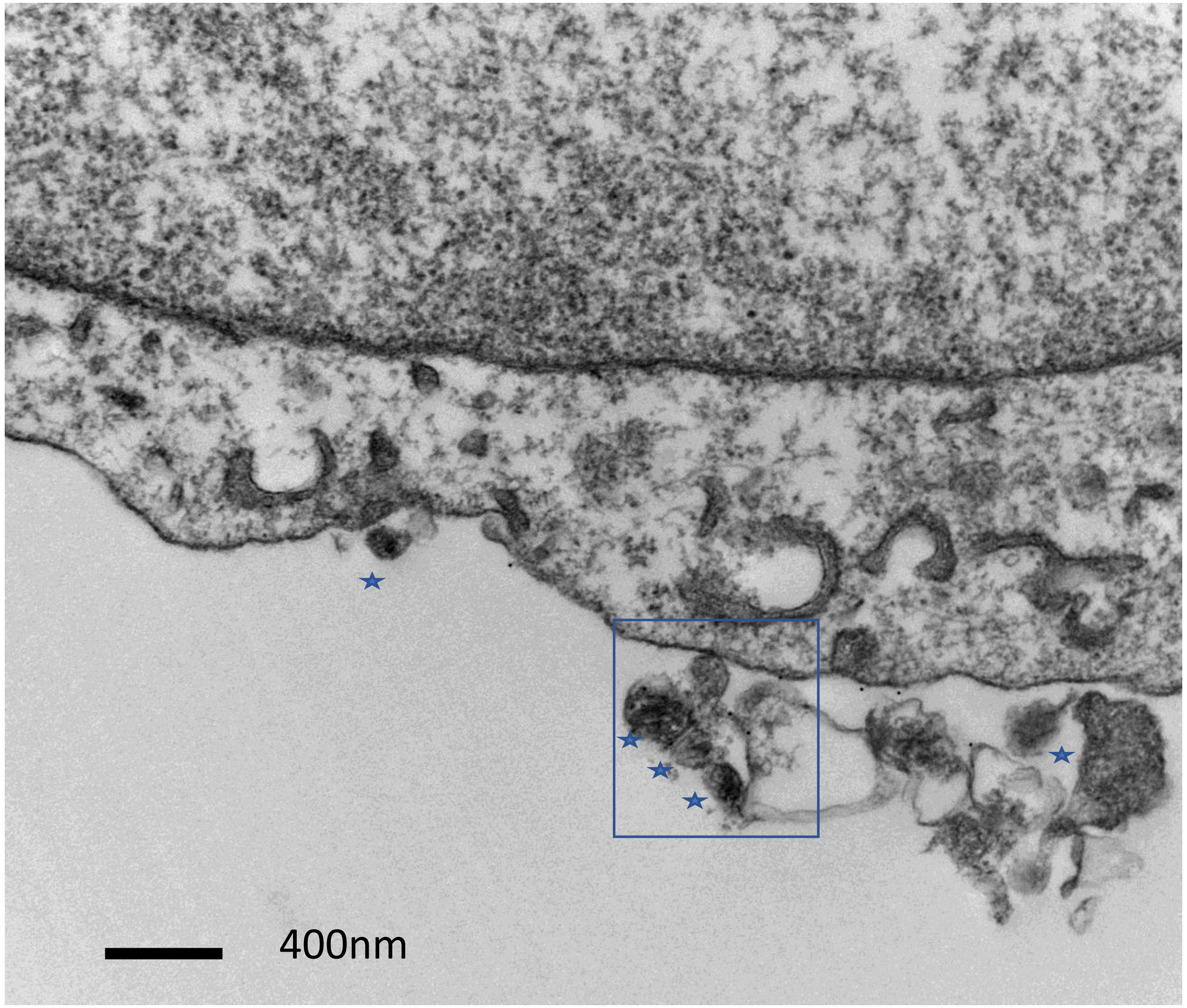

Supplemental Figure 7. TEM images of anti-CD62L immunogold labeled uninfected cells in the presence of DMSO (A) or BB-94 (B). The scale bar represents 200nm. C) Majority of gold particles labeled cell surface CD62L in infected cells (black arrows). D,E) gold particle-labled CD62L associated with budding virions (D) and released virions (E). F) A TEM image of anti-CD62L immunogold labeled HIV-1$_{LAI}$ infected CD4 T cell in the presence of BB-94. The image is a slightly lower resolution image of Figure 7B left panel. The boxed area is presented as a magnified view in Figure 7B. Virions with visible capsid are marked with blue stars. The TEM image also shows several virions without anti-CD62L immunogold labeling. The scalebar is 400nm.

Supplemental Figure 8

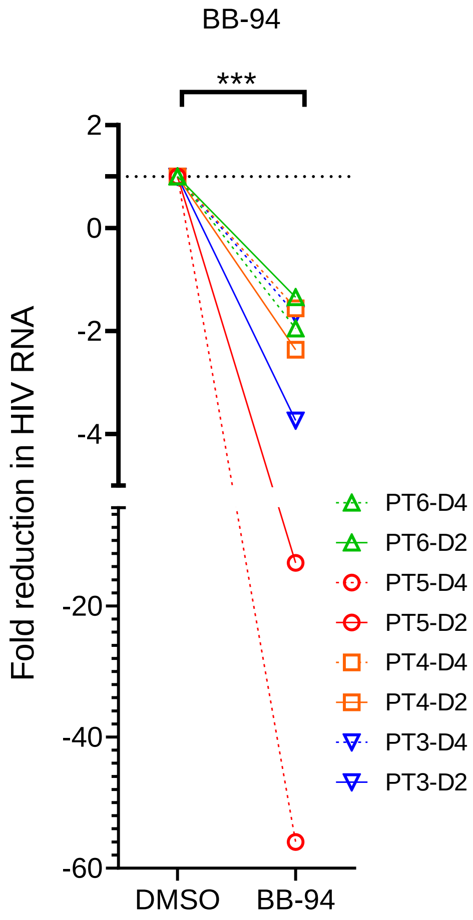

Supplemental Figure 8. Inhibition of BB-94 to HIV-1 viral release in CD4 T cells from HIV positive individuals. The detected HIV viral RNA copy numbers/ml in the presence of BB-94 are normalized against their respective DMSO control samples and displayed as fold reduction of the controls. p-values are * <0.05, **<0.01, *** <0.0005.
